# Highly Crowded Twisted Thienylene‐Phenylene Structures: Evidence for Through‐Space Orbital Coupling in a [4]Catenated Topology

**DOI:** 10.1002/advs.202105785

**Published:** 2022-02-08

**Authors:** Tanja Desirée Leitner, Jan Simon von Glasenapp, Rainer Herges, Elena Mena‐Osteritz, Peter Bäuerle

**Affiliations:** ^1^ Institute of Organic Chemistry II and Advanced Materials University of Ulm Albert‐Einstein‐Allee 11 Ulm 89081 Germany; ^2^ PPG Industries Lackfabrik GmbH Erlenbrunnenstraße 22 Bodelshausen 72411 Germany; ^3^ Otto Diels‐Institute of Organic Chemistry Christian‐Albrechts University Kiel Otto‐Hahn‐Platz 4 Kiel 24098 Germany

**Keywords:** ACID calculation, thienylene‐phenylenes, through‐space conjugation, toroidal p*_z_*‐orbital coupling, X‐ray structure analysis

## Abstract

Sterically highly crowded and twisted thienylene‐phenylenes are synthesized and structurally characterized. Single‐crystal X‐ray structure analyses and theoretical studies give evidence of through‐space delocalization of *π*‐electrons of peripheral (hetero)aromatic rings in toroidal and catenated topology.

## Graphical abstract

Sterically highly crowded and twisted thienylene‐phenylenes are synthesized and structurally characterized. Single‐crystal X‐ray structure analyses and theoretical studies give evidence of through‐space delocalization of *π*‐electrons of peripheral (hetero)aromatic rings in toroidal and [4]catenated topology (light blue rings).

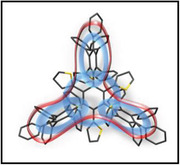

## 1 Introduction

**Scheme 1:**
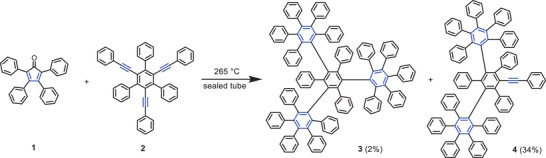
Synthesis of highly crowded polyphenylene dendrimer **3** and by‐product **4** by multiple Diels–Alder cycloaddition.

3D polyphenylene dendrimers and nanostructures are not only of great importance due to their wide range of potential applications as nanomaterials, but are also attractive for their challenging synthesis and great aesthetics. A frequently used basic structural element is represented by hexaphenylbenzene, which readily can be synthesized by Diels–Alder cycloaddition of tolane and tetracyclone in high yields. Müllen and co‐workers took this concept to build up myriads of highly crowded and structurally defined polyphenylenes with sizes up to the 10 nm regime.^[^ ^1^ ^]^ Subsequent cyclodehydrogenation reactions of these dendritic 3D structures allowed for the preparation of large, molecularly defined 2D polycyclic aromatic hydrocarbons, also called nanographenes, with the largest hexagonal disk including 222 carbons.^[^ ^2^ ^]^ More recently, the sophisticated concepts were used by the Müllen group to prepare to date inconceivable structures of defined graphene nanoribbons as a new family of carbon‐based semiconductors via solution and on‐surface chemistry. In contrast to graphene, the band gap in these nanoribbons is opened due to the geometric confinement and therefore can be applied in electronic application such as switches and transistors.^[^ ^3^ ^]^ A highly challenging project in this category was the synthesis of “phenylogous” model compounds for cubic graphite as hypothetical carbon allotrope which fascinates due to an exceptional 3D structure entirely comprising benzene rings, from which each of them is connected to six other rings. Such a model and precursor subunit of “phenylogous” cubic graphite with the extended hexaphenylbenzene structure **3** was formed in a threefold [4+2]‐cycloaddition of tetracyclone **1** and triply ethynylated quaterphenyl **2** under harsh reaction conditions and isolated in 2% yield besides the doubly reacted product **4** (34% yield) (**Scheme** **1**).

A single‐crystal X‐ray structure analysis of **3** revealed the expected threefold symmetry and a high packing density of the overcrowded molecule.^[^ ^4^ ^]^

**Figure 1:**
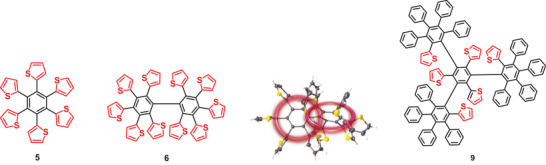
Twisted thienylene‐phenylenes **5** (left) and **6** (middle left) gave evidence of through‐space delocalization of *π*‐electrons of peripheral (hetero)aromatic rings in toroidal and [2]catenated topologies for neutral molecules (middle right). Targeted trigonal thienylene‐phenylene analog **9** of polyphenylene **3** (right).

In this respect, we have described more recently the development of sterically crowded and twisted thienylene‐phenylenes and compared them to their polyphenylene counterparts. The replacement of benzene rings by thiophenes in hexa(2‐thienyl)benzene **5** and deca(2‐thienyl)biphenyl **6** also revealed steric hindrance and propeller‐like arrangement of the peripheral thiophene rings, but reduced steric congestion and distortion effects due to the smaller geometric extension of thiophene compared to benzene. Interestingly, through‐space *π*‐conjugation of the *ipso*‐carbons in a toroidal and catenated topology has been evidenced from X‐ray structure analysis and theoretical calculations (anisotropy of the induced current density (ACID)) (**Figure** **1**). Furthermore, the electron‐rich thiophene units in **5** and **6** exerted the expected influence on the electronic properties.^[^ ^5^ ^]^

In this work, we now present the advancement of this structural concept by replacing six benzene rings in polyphenylene dendrimer **3** by corresponding thiophene units to result in targeted trigonal thienylene‐phenylene **9** (Figure 1). Structural analyses of the target molecule give evidence of toroidal through‐space delocalization of *π*‐electrons in a [4]catenated topology.^[^ ^6^ ^]^ Furthermore, the cyclotrimerization reaction of butadiyne **7** was studied in more detail leading to interesting structures.

## 2 Results and Discussion

### 2.1 Synthesis of Thienylene‐Phenylene Dendrimer **9**

**Scheme 2:**
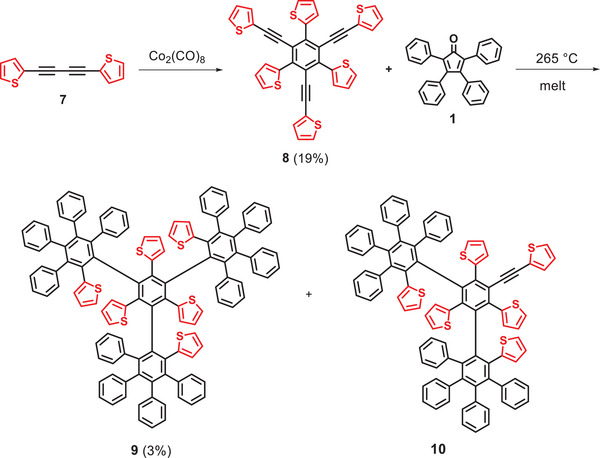
Synthesis of highly crowded thienylene‐phenylene dendrimer **9** and **10** by multiple Diels–Alder cycloaddition of precursor **8** and tetracyclone **1**.

The preparation of crowded thienylene‐phenylene **9** is illustrated in **Scheme** **2**. 1,4‐Di(thien‐2‐yl)buta‐1,3‐diyne **7^[^** ^7]^ and tetracyclone **1^[^** ^8]^ were synthesized following modified literature procedures. The subsequent cobalt‐mediated cyclotrimerization of diyne **7** in 1,4‐dioxane at 110 °C gave hexasubstituted benzene derivative **8** as thienyl analog of **2** in a moderate yield of 19%. The desired thienylene‐phenylene **9** was subsequently obtained by threefold Diels–Alder cycloaddition of precursor **8** and excess tetracyclone **1** at 265 °C in the melt. Purification of target dendrimer **9** was achieved by repeated column chromatography and recycling high‐pressure liquid chromatography (HPLC) and the pure product was collected in 3% yield as colorless solid. Increased reaction temperature had negative impact and did not lead to higher yields, because the substrates started increased decomposition. As the main product, the doubly reacted **10** was isolated in 54% yield by column chromatography.

The molecular structure of dendrimer **9** was assigned by high‐resolution mass spectrometry (HRMS), which showed the intense molecular‐ion peak at *m*/*z* = 1711.4489 (Figure S1, Supporting Information), ^1^H and ^13^C NMR spectroscopy. In the ^1^H NMR spectrum, an unusually high‐field‐shifted multiplet signal (*δ* = 5.38–5.28 ppm, integral of three) for the *β*‐protons in 3‐position of the inner three thiophenes is observable (Figure S2, Supporting Information). This shielding effect was also observed for twisted thienylene‐phenylenes **5** and **6^[^** ^5]^ and reference molecule **3^[^** ^4]^ and is induced by aromatic ring current arising from the preferred conformation, in which these *β*‐protons are lying above and beneath the center of the perpendicularly arranged phenylene rings. Due to the very low solubility of **9**, a ^13^C NMR spectrum was measured in C_2_D_2_Cl_4_ as solvent at 373 K and showed 37 signals for 120 carbons indicating the expected *D_3h_* symmetry of the molecule. Single‐crystal X‐ray structure analyses of precursor **8** and target derivative **9** gave absolute certainty of the structures and the regioregularity of the substituents (vide infra).

**Scheme 3:**
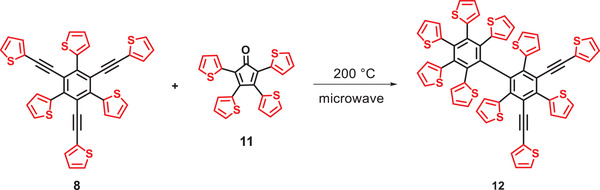
Diels–Alder cycloaddition of precursor **8** with tetra(2‐thienyl)cyclopentanone **11**.

The original attempts to synthesize a thienylene‐phenylene dendrimer analogous to **9** but with an increased ratio of thiophene rings by similar reaction of precursor **8** and tetra(2‐thienyl)cyclopentanone **11** resulted in only the mono‐reacted derivative **12** in low yield (**Scheme** **3**, HRMS in Figure S3, Supporting Information). Due to the lower thermal stability of cyclopentadienone **11^[^** ^5]^ as compared to **1**, lower reaction temperatures of up to 200 °C were possible and therefore additional Diels–Alder cycloadditions to the desired dendrimer were hampered.

### 2.2 Structural Characterization of Thienylene‐Phenylenes **8** and **9** by Single‐Crystal X‐Ray Structure Analysis

The molecular structures of the novel twisted thienylene‐phenylene precursor **8** and targeted **9** were investigated and further corroborated by single‐crystal X‐ray structure analyses.

**Figure 2:**
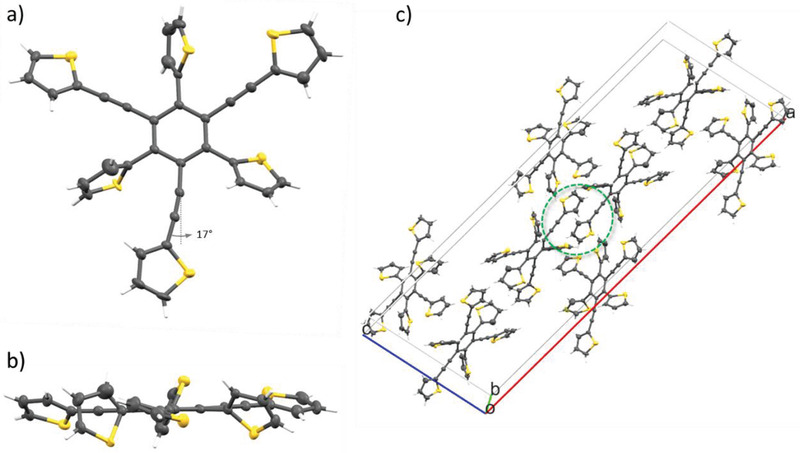
Molecular structure of hexasubstituted benzene **8**: a) front view, including angle of deviation from linearity of the ethynylene bridge, b) side view, and c) unit cell containing eight molecules. *π*−*π* Intermolecular interactions perpendicular to the [10 −4 −7] plane are labelled in (c) (green circle). Only one of two disordered molecules is illustrated. Ellipsoids were depicted at 50% probability in all cases (*R* factor 3.45%, CCDC number: 1884748).

Single crystals of 2,2′,2″‐{[2,4,6‐tri(thien‐2‐yl)benzene‐1,3,5‐triyl]tris(ethyne‐2,1‐diyl)}trithiophene **8** were obtained as colorless needles by slow evaporation from dichloromethane (DCM) solution. The molecule crystallizes in the monoclinic space group *C2/c* with eight molecules in the unit cell (**Figure** **2**c). The lattice parameters amount to *a* = 50.14(2) Å, *b* = 7.352(3) Å, *c* = 16.854(8) Å, and *α* = 90°, *β* = 100.86(2)°, *γ* = 90° (Table S1a, Supporting Information).

The molecular structure of **8** presents disorder in five of the six thiophene rings due to the possibility of syn‐anti arrangment whereby keeping the same molecular volume. Higher torsion angles between 48.0(2)° and 81.3(2)° were identified for the thiophenes directly attached to the central benzene ring, whereas the thiophenes connected through the ethynylene bridge are closer to planarity by 18.4(2)° to 27.1(2)° (Figure 2a,b and Figure S10a and Table S1d, Supporting Information). Furthermore, a bending of the acetylene bridge up to 17° can be observed in one of the substituents (Figure 2a and Table S1c, Supporting Information). We have to point out that no packing effects are responsible for this flexibility of the ethynylene bridge as no short contacts to the involved C‐atoms were found. Also in this case, a bond length equalization within the central benzene ring with mean value of bond alternance of 0.003 Å was observed. The bond distances between the benzenoid carbons and the *ipso*‐C atoms of the adjacent thiophenes range from 1.479(3) to 1.486(3) Å. These distances are undercut by the ones to the *ipso*‐C atoms of the ethynyl bridges which lie between 1.433(3) and 1.437(3) Å (Figure S10a and Table S1b, Supporting Information). The unit cell of hexasubstituted benzene **8** contains eight molecules and *π*−*π* interactions between molecules can be observed at the ethynylene‐thiophene arms at distances of 3.473 Å (Figure 2c, green circle). Further intermolecular interactions appear between H—C and S—S atoms at distances far below van de Waals radii. The molecules are ordered in planes and interact in a herringbone arrangement with up to seven neighboring molecules predominantly via S—S interactions (Figure S10b and Table S1e, Supporting Information). Intramolecular short contacts occur between the *ipso‐*C atoms and also between carbon and sulfur atoms. All of them are of relevance since the overlap of *π*‐orbitals should occur at distances below van der Waals radii which indicates potential *π*‐electron delocalization that extends over the entire molecule in a toroidal topology (Figure S10b,c, Supporting Information).^[^ ^5^ ^]^

**Figure 3:**
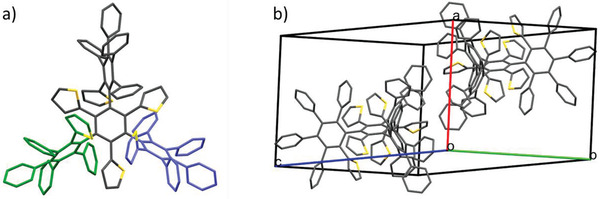
a) Molecular structure of target compound **9** and b) unit cell containing two molecules. For clarity, only one of the two disordered molecules at each site is illustrated and ellipsoids and hydrogen atoms are omitted. Two of three pentaarylbenzene units in (a) have been colored in green and blue as a guide to the eye (*R* factor = 8.08%, CCDC number: 1884754).

Single crystals of dendrimer **9** were obtained by slow diffusion of *n*‐hexane into a DCM solution. **9** crystallizes as DCM adduct in the triclinic space group $P\bar{1}$ with two molecules in the unit cell (Figure 6b). The lattice parameters amount to *a* = 13.0074(6) Å, *b* = 8.8041(7) Å, *c* = 20.8851(9) Å, and *α* = 104.484(4)°, *β*  = 97.403(4)°, *γ* = 92.008(4)° (Table S1a, Supporting Information). All three thiophenes, which are directly attached at the central benzene ring, show orientational disorder. Disorder is also found at the pentaarylbenzene units. The disorder causes two possible conformers from which only one is shown in **Figure** **3**a. The thiophenes directly attached at the central benzene ring show smaller torsion angles (40.5°(4) to 45.0°(4)) than the corresponding inner phenyl substituents (69°(1) to 76.7°(9)). Their phenyl and thienyl substituents are as well propeller‐like distorted with angles of 51°(3) to 81°(3) (Figure S11 and Table S2c, Supporting Information). Bond distances between the central phenyl ring and the three phenyl residues account to 1.501–1.515 Å and 1.486–1.507 Å to the directly attached thiophene subunits (Table S2b, Supporting Information). These bond lengths are remarkably longer than the 1.44 Å observed in strain‐free 2,5‐diphenylthiophene and are addressed to the high steric congestion in **9**.^[^ ^16^ ^]^ The highly crowded dendritic 3D structures of **9** obviously led to strong bond length alternation in peripheral benzene rings whereas the central unit shows the expected bond length equilibration with deviations as low as 0.001 Å.

**Figure 4:**
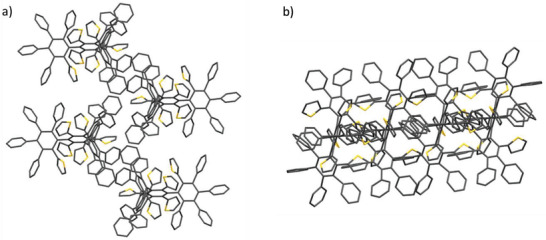
Packing mode of dendrimer **9** a) perpendicular and b) parallel to the [1 0 0] plane. Ellipsoids and hydrogen atoms have been omitted for clarity.

The described molecular arrangement results in an intertwined quadruple propeller. In the bulk, the molecules are packed in perfectly aligned rows perpendicular to the [1 0 0] plane, stabilized by only two intermolecular H—C interactions. Between the individual rows, which alternately face toward each other, further H—C interactions occur (**Figure** **4** and Table S2d, Supporting Information). As expected, no *π*−*π* stacking was found in the highly distorted structure. From the 22 aromatic rings in the molecule, half of them undergo up to 13 intermolecular interactions, most of them are C—H contacts involving six surrounding molecules (Table S2d, Supporting Information).

**Figure 5:**
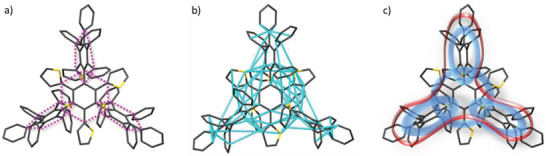
Intramolecular short contacts for dendrimer **9**: short contacts between the *ipso*‐C atoms are separately labeled with magenta dotted lines in (a). The C—C, C—S, and S—S heavy atom short contacts are labeled with cyan dotted lines in (b). c) The resulting multiply catenated topology is sketched with blue circles and the peripheral pathway with a continuous red ribbon. In all cases, hydrogen atoms and ellipsoids have been omitted for clarity.

A much higher number of intramolecular short contacts far below the sum of the van der Waals radii between *ipso*‐C atoms and between C—C, C—S, and S—S atoms have been found for **9** compared to precursor **8** which clearly evidences the highly crowded 3D structure filling three times more volume (**Figure** **5**a,b and Table S2e,f, Supporting Information). The paths of interacting atoms in **9** causing potential overlap of their p*_z_*‐orbitals resemble four intertwined rings, in which through‐space conjugation of *π*‐electrons in a [4]catenated topology occurs (blue circles in Figure 5c). In addition, based on the interactions involving heavy atoms of the peripheral (hetero)aryl rings, a second pathway of overlapping p*_z_*‐orbitals can be sketched connecting all 15 outer aromatic rings (red ribbon in Figure 8c).

### 2.3 Further Investigation of the Cyclotrimerization of Butadiyne **7**

**Figure 6:**
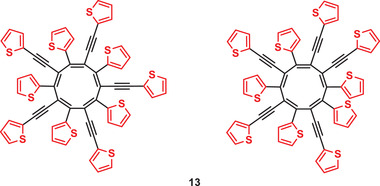
Two regioisomeric structures of cyclopentamer **13**, which arise from various possibilities of head‐to‐tail and head‐to‐head couplings, isolated from the cobalt‐catalyzed cyclomerization of butadiyne **7**.

We noted for the cobalt‐catalyzed reaction of butadiyne **7** in 1,4‐dioxane at 110 °C that not only the desired hexasubstituted benzene **8** as cyclotrimer was obtained in moderate yield, but also a series of higher molecular mass products was formed during the reaction which was detected by HRMS in the raw product mixture (Figure S4, Supporting Information). Mass peaks, which correspond to higher cyclooligomerization products consisting of 5–10 monomer units of **7** were identified. Subsequent multiple chromatographic purification provided fractions, which showed an intense molecular ion peak at *m*/*z* = 1069.9550 in HRMS proving cyclopentameric structures (Figure S5, Supporting Information). The NMR spectra imply the formation of regioisomeric [10]annulenes **13** (**Figure** **6** and Figure S6, Supporting Information), but the unequivocal assignment of such structures could not be accomplished due to the variable head‐to‐tail and head‐to‐head couplings of monomeric butadiyne **7** leading to the formation of a series of regioisomers.^[^ ^9^ ^]^

We assume that the mechanism for the formation of such sterically crowded cyclopentamers is analogous to the insertion mechanism of the cyclotrimerization to **8** (Scheme S1, Supporting Information). The formation of larger conjugated macrocyclic oligo‐ and polyacetylenes, which can be regarded as large [n]annulenes, has been recently described by Veige et al. for the ring‐expansion polymerization of phenylacetylene or acetylene with tungsten metathesis catalysts.^[^ ^10^ ^]^

Cyclooligomerizations of alkynes go back to 1948, and both, the Ni‐catalyzed formation of substituted benzenes via [2+2+2]‐cycloaddition^[11]^ and cyclooctatetraene (COT, [8]annulene) via [2+2+2+2]‐cycloaddition,^[12]^ was introduced by Reppe et al. and has been intensively investigated since then. Besides nickel, diverse transition metals such as cobalt, rhodium, palladium, ruthenium, and others were used^[^ ^13^ ^]^ and the general mechanism of the catalytic cycle was established.^[^ ^13^ ^b,^ ^14^ ^]^ With this background, we have alternatively applied the Wilkinson catalyst [RhCl(PPh_3_)_3_] for the cyclooligomerization of butadiyne **7**. As in the case of Co_2_(CO)_8_ as catalyst, hexasubstituted benzene **8** was isolated as main product in a moderate yield of 10% besides cyclopentamers **13** after tedious chromatographic work‐up. However, an additional fraction comprising a tetrameric product was isolated in 2% yield which in HRMS showed a molecular ion peak at the mass of a cyclotetramer (*m*/*z* = 855.96114) (Figure S7, Supporting Information). The expected COT core could not be assigned in the ^1^H NMR spectra, because the spin system of only seven 2‐thienyl rings of eight was detected (Figure S8, Supporting Information). However, H,H‐COSY‐NMR experiments revealed three additional signals, which pointed to a 2,3‐substituted thiophene ring and an additional phenylic proton (Figure S9, Supporting Information).

**Figure 7:**
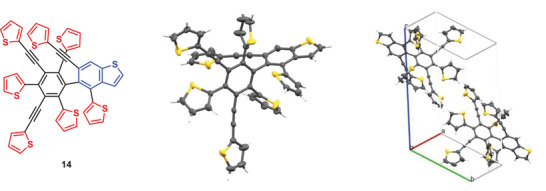
Molecular structure of thienylene–phenylene **14** (left, middle). Unit cell with the two molecules (right). For clarity, only one of the two disordered molecules (*cis*‐*trans* isomerization at certain thiophene rings) is illustrated. Ellipsoids were depicted at 50% probability in all cases (*R* factor 4.57%, CCDC number: 1884744).

**Figure 8:**
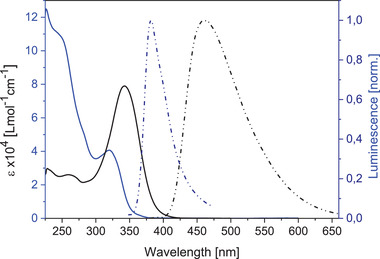
UV/vis absorption spectrum of target dendrimer **9** (blue line, *c*  ≈  5 ×  10^–6^ mol L^−1^) and precursor **8** (black line, *c*  ≈  5 ×  10^–6^ mol L^−1^) and normalized emission spectrum of **9** (blue dashed line, excitation at 250 nm, *c*  ≈  1 ×  10^–7^ mol L^−1^) and precursor **8** (black dashed line, excitation at 350 nm, *c*  ≈  1 ×  10^–7^ mol L^−1^), measured in DCM at room temperature.

The molecular structure was finally resolved by single‐crystal X‐ray structure analysis (Figure 8, vide infra) revealing the entirely unexpected hexasubstituted thienylene‐phenylene **14**, in which besides three 2‐thienyl and two 2‐thienylethynyl groups, the central benzene ring is substituted by a benzo[*b*]thiophene unit bearing a single thiophene in 4‐ and a thienylethynyl group in 6‐position (**Figure** **7**, left). With this information, the ^1^H NMR spectrum of **14** was fully assignable and in accordance with the structure.^[^ ^9^ ^]^ The reaction mechanism to the unique formation of the benzo[b]thiophene ring is rather speculative at the moment. We assume that the product arises from an Rh‐catalyzed cycloaddition of butadiyne **7** and a single thienylethynyl substituent of the main cyclotrimerization product **8** under the formation of an intermediate cyclopentadiene rhodium complex. Insertion, 1,3‐proton shift, and final reductive elimination of the metal should result in thienylene‐phenylene **14** (Scheme S2, Supporting Information).

Colorless single crystals of 5‐(thien‐2‐yl)‐4‐(thien‐2‐ylethynyl)‐6‐[2,4,6‐tri(thien‐2‐yl)‐3,5‐bis(thien‐2‐ylethinyl)phenyl]benzo[*b*]thiophene **14** were obtained by slow evaporation from DCM solution and crystallizes in the triclinic space group *P* $\bar{1}$ with two molecules in the unit cell (Figure 7, right). The lattice parameters amount to *a* = 9.5703(4) Å, *b* = 11.4821(4) Å, *c* = 19.3739(5) Å, and *α* = 102.118 (3)°, *β* = 96.605(3)°, *γ* = 98.016(3)°. In the refinement of the X‐ray structure, disorder due to syn‐anti‐isomerization in six thiophene rings has been found. Key parameters of the crystal structure refinement are summarized in Table S1 (Supporting Information).

The molecular structure of **14** is shown in Figure 7 (middle) and the geometry points to some interesting features: the central benzene ring evidences normal bond equalization with a mean value of bond alternance of 0.007 Å, whereas the single and double bonds in the benzene ring of the benzo[*b*]thiophene unit are localized with mean bond alternance of 0.03 Å which is typical for benzo[*b*]thiophenes (Figure S12a and Table S3b, Supporting Information).^[^ ^15^ ^]^ The thiophenes directly attached to the central benzene ring are distorted out of the plane by 55.5°, 76.1°, and 83.3° and the benzo[*b*]thiophene moiety is almost perpendicular (87.9°) which indicates lack of conjugation between the aromatic rings as a consequence of the crowded structure. On the contrary, the ethynyl‐bridged thiophenes remain closer to planarity (17.1° and 25.4°) with respect to the central benzene ring (Table S3d, Supporting Information). All three ethynylene bridges show a certain flexibility with deviation from planarity of 4° to 11°, whereby that connected to the benzo[*b*]thiophene unit is the most bent (Table S3c, Supporting Information). Despite the lack of symmetry of **14**, the molecules order in planes with almost no mutual intermolecular interactions, but interact with molecules in adjacent planes. In fact, each molecule of **14** interacts at distances below van der Waals radii with eight surrounding molecules via C—S, H—C, and H—S contacts (Figure S12b and Table S3e, Supporting Information). Interesting intramolecular interactions have been found as well: in addition to the short contacts between *ipso*‐C atoms, up to 13 further C—C, C—S, and S—S interactions were detected and underline the compactness of the molecular structure of **14** (Figure S12c and Table S3f, Supporting Information).

### 2.4 Optical and Electrochemical Properties

The optoelectronic properties of twisted phenylene‐thienylene precursor **8** and derivative **9** were investigated by UV‐vis absorption and fluorescence spectroscopy and by cyclic voltammetry. The data are collected in **Table** **1**, optical spectra are shown in **Figure** **8** and cyclic voltammograms in Figure S13 (Supporting Information). Similar trends were found compared to sterically crowded thienylene‐phenylenes **5** and **6**.^[^ ^5^ ^]^ In comparison to the polyphenylenes, the electron‐rich thiophene units in derivatives **8** and **9** exert bathochromic shifts in absorption and emission and decrease the oxidation potential in electrochemistry. The same effect is noted among the two molecules: due to the highly twisted topology of branched dendrimer **9**, *π*‐conjugation of the (sub)chromophores is reduced leading to hypsochromically shifted absorption and emission compared to the more planar **8**.

**Table 1:** Optical and electrochemical data of phenylene‐thienylene derivative **9** and precursor **8**

	*λ*_abs_ [nm]^a)^	*E*_g_^opt^ [eV]	*λ*_em_ [nm]^b)^	*E*_p_^ox1^ [V]	*E*_p_^ox2^ [V]	*E*_HOMO_ [eV]	*E*_LUMO_ [eV]^c)^
**8**	228, 259, 343	3.21	460	(≈0.96)^d)^	‐	−6.06	−2.85
**9**	226, 247 (s), 280 (s), 320	3.52	382	0.91	1.13	−5.95	−2.43

^a)^ Measured in DCM, maximum in italics, (s) denotes shoulder;

^b)^ Measured in DCM;

^c)^ Calculated with *E*_g_^opt^;

^d)^ Oxidation onset, no clear wave visible probably due to polymerization.

### 2.5 Theoretical ACID Calculations on Thienylene‐Phenylene Dendrimer **9**. Evidence for Toroidal Through‐Space Conjugation in a [4]Catenated Topology

**Figure 9:**
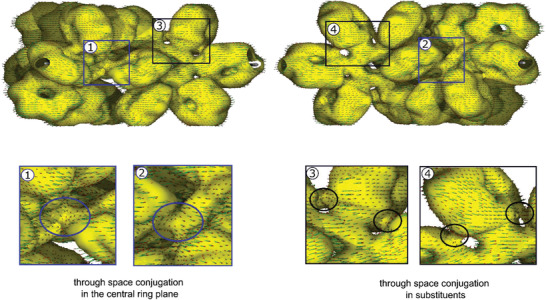
ACID plot of dendrimer **9** at an isovalue of IV = 0.009. The magnetic field is orthogonal to the central benzene ring. To visualize the through‐space conjugation in the central benzene plane, the ACID hypersurface is shown from two different viewpoints to present both sides of the thiophene substituent. The current density is plotted as green arrows onto the isosurface. The length of the arrows is proportional to the current density. The wavefunction was calculated based on crystal structure at a B3LYP/6‐31G** level of theory. The current density field was computed using the option IOp (10/93 = 1) implemented in Gaussian 16.^[^ ^18^ ^]^ The *ipso* through‐space conjugation is clearly visible for the central plane and the substituents, however no diatropic ring current following the *ipso* conjugation pathway was observed.

For investigation of electron delocalization, our ACID method was employed.^[^ ^17^ ^]^ The ACID scalar field is interpreted as the density of delocalized electrons and can be plotted as an isosurface (yellow surfaces in **Figure** **9**).

For the sake of simplicity and to save computer time, the conformation **9** obtained from X‐ray structure analysis was analyzed. The expected strong *π* delocalization is clearly visible for benzene and thiophene. Beyond the classic aromatic ring delocalization, there is distinct through‐space delocalization between the rings. In this respect, the six *ipso* carbon atoms in each of the tetraphenyl‐thienyl‐phenyl dendrons exhibit through‐space conjugation (Figure 9, ③ and ④) and the same was observed for the *ipso* carbon atoms of aromatic rings adjacent to the central benzene ring (Figure 9, ① and ②). Hence, the ACID analysis strikingly confirms the topology of through‐space interaction shown in Figure 5.

## 3 Conclusions

In summary, we realized synthesis and characterization of dendritic, trigonal thienylene‐phenylene **9** with a highly crowded 3D structure comprising 16 phenyl and 6 thiophene rings. Structural analyses of the target molecule by single‐crystal X‐ray structure analysis and calculation of electron delocalization with the ACID method strikingly revealed toroidal through‐space delocalization of *π*‐electrons in a [4]catenated topology. The paths of interacting *ipso* carbon atoms in **9** causing potential overlap of their p*_z_*‐orbitals resemble four intertwined rings. This finding extends the previously described toroidal and [2]catenated through‐space conjugation in corresponding biphenyl **6**.^[^ ^5^ ^]^ Additionally, we further investigated the cyclotrimerization reaction of butadiyne **7** and found interesting byproducts, which were characterized by NMR spectroscopy and single‐crystal X‐ray structure analysis. Among them, we could isolate unprecedented cyclopentamers **13** and benzo[*b*]thiophene‐containing thienylene‐phenylene **14**.

## 4 Experimental Section

### Materials and Methods

1,4‐Di(*thien*‐2‐yl)buta‐1,3‐diyne **7**,^[^ ^7^ ^]^ tetracyclone **1**,^[^ ^8^ ^]^ and 2,3,4,5‐tetra(2‐thienyl)cyclopenta‐2,4‐dien‐1‐on **13^[^** ^19]^ were prepared according to literature. Dried tetrahydrofuran (THF, Sigma Aldrich) was used from an MB SPS‐800 solvent purification system (MBraun). 1,4‐Dioxane (Merck) was dried over CaH_2_ and distilled prior to use. DCM, methanol, petroleum ether (PE), and *n*‐hexane were purchased from VWR and distilled prior to use. *n*‐Butyl lithium (BuLi, 1.6 m in *n*‐hexane) was purchased from Acros Organics, Co_2_(CO)_8_ from Sigma Aldrich. Thin‐layer chromatography was performed on aluminum plates, precoated with silica gel, Merck Si60 F254. Preparative column chromatography was carried out on glass columns packed with silica gel, Merck Silica 60, particle size 40–63 µm. HPLC was performed on a Shimadzu CBM‐20A equipped with an SPD‐20A UV/VIS detector and an LC‐8A solvent system with a Macherey‐Nagel column (Nucleosil 100–5 NO_2_). NMR spectra were recorded on a Bruker Avance 400 (^1^H NMR: 400 MHz, ^13^C NMR: 101 MHz) at 293 K. Chemical shift values (*δ*) were given in parts per million (ppm) and were calibrated on residual nondeuterated solvent peaks (^1^H NMR: *δ*_H_ = 5.32 for CD_2_Cl_2_, *δ*_H_ = 7.26 for CDCl_3_, *δ*_H_ = 3.58 for THF‐d_8_, *δ*_H_ = 5.98 for TCE‐d_2_; ^13^C NMR: *δ*_C_ = 54.0 for CD_2_Cl_2_, *δ*_C_ = 77.2 for CDCl_3_, *δ*_C_ = 73.7 for TCE‐d_2_) as an internal standard. The splitting patterns were labeled as follows: s (singlet), d (doublet), dd (doublet of doublet), and m (multiplet). Melting points were measured using a Büchi Melting Point M‐565. High‐resolution mass spectra (HRMS, MALDI‐FTICR, and APCI) were recorded on a Bruker Solarix (Bruker Daltonik GmbH), using *trans*‐2‐[3‐(4‐*tert*‐butylphenyl)‐2‐methyl‐2‐propenylidene]malononitrile (DCTB) as a matrix. Elemental analyses were performed on an Elementar Vario EL. UV/Vis absorption spectra were recorded on a Perkin Elmer Lambda 19 spectrometer. Fluorescence spectra were recorded on a Perkin Elmer LS 55 Luminescence spectrometer. Cyclic voltammetry experiments were carried out using a computer‐controlled Autolab PGSTAT30 potentiostat in a three‐electrode single‐compartment cell with a platinum working electrode, a platinum wire counter electrode, and a Ag/AgCl reference electrode. All potentials were internally referenced to the ferrocene/ferricenium couple. The diffraction data of crystals of **8**, **9**, and **12** were collected in a stream of nitrogen at 180 K on an Agilent SuperNova, Cu at zero, Atlas CCD using graphite‐monochromated Cu K*α* radiation. Data collection strategy was performed with the APEX2 software, data reduction, absorption correction, and cell refinement with CrysAlisPro 171. The structure was solved by direct methods with SHELXL‐2016/6, revealing all atoms of the derivatives. H atoms were discernible from difference Fourier maps during refinement on F2 with SHELXL‐97. For the final model, all atoms were refined anisotropically.

### Synthetic Methods

*2,2′,2″‐{[2,4,6‐Tri(thien‐2‐yl)benzene‐1,3,5‐triyl]tris(ethyne‐2,1‐diyl)}trithiophene* ***8***: 1,4‐Di(thien‐2‐yl)buta‐1,3‐diyne **7** (400 mg, 1.87 mmol) and Co_2_(CO)_8_ (135 mg, 0.39 mmol) in 20 mL abs. 1,4‐dioxane were stirred at 110 °C for 2 days. The solvent was removed and the residue was purified by column chromatography (PE/DCM 3:1). Phenylene‐thienylene **8** (77 mg, 1.20 mmol, 19%) was obtained as light‐yellow solid after twofold recrystallization from methanol and *n*‐hexane/DCM. M.p. 276–279 °C;^[^ ^1^ ^]^ H NMR (400 MHz, THF‐d_8_): *δ* 7.63 (dd, ^3^ *J*_(H,H)_ = 5.1 Hz, ^4^ *J*_(H,H)_ = 0.9 Hz, 3H, *α‐H^5^‐Th*), 7.40–7.37 (m, 6H, *α‐H^5′^‐Th*, *β‐H^3^‐Th*), 7.19 (dd, ^3^ *J*_(H,H)_ = 5.0, 3.6 Hz, 3H, *β‐H^4^‐Th*), 6.92 (dd, ^3^ *J*_(H,H)_ = 5.0, 3.7 Hz, 3H, *β‐H^4′^‐Th*), 6.87 (dd, ^3^ *J*_(H,H)_ = 3.6, ^4^ *J*_(H,H)_ = 1.0 Hz, 3H, *β*‐*H^3′^‐Th*) ppm; ^13^C NMR (101 MHz, CD_2_Cl_2_): *δ* 139.5, 138.8, 132.8, 130.1, 128.9, 127.7, 127.3, 127.0, 125.6, 123.1, 92.8, 91.7 ppm; HRMS (MALDI‐FTICR) *m*/*z*: calcd. for C_52_H_30_S_10_: 641.97273; found 641.97251 [M]^+^ (*δm*/*m* = 0.7 ppm), 892.11948 [M+DCTB]^+^, 1142.26549 [M+2DCTB]^+^; CCDC 1884748.

Derivatives of cyclopentamers **13** were obtained as side products and were separated by HPLC (recycling mode, *n*‐hexane/DCM 3:2). Isolated derivative A: ^1^H NMR (400 MHz, CD_2_Cl_2_): *δ* 7.58 (dd, ^3^ *J*_(H,H)_ = 5.1 Hz, ^4^ *J*_(H,H)_ = 1.2 Hz, 1H, *α‐H^5^‐Th*), 7.45 (dd, ^3^ *J*_(H,H)_ = 3.6 Hz, ^4^ *J*_(H,H)_ = 1.2 Hz, 1H, *β‐H^3^‐Th*), 7.42 (dd, ^3^ *J*_(H,H)_ = 5.0 Hz, ^4^ *J*_(H,H)_ = 1.2 Hz, 1H, *α‐H^5^‐Th*), 7.37 (dd, ^3^ *J*_(H,H)_ = 5.0 Hz, ^4^ *J*_(H,H)_ = 1.2 Hz, 2H, *α‐H^5^‐Th*), 7.34 (dd, ^3^ *J*_(H,H)_ = 4.9 Hz, ^4^ *J*_(H,H)_ = 1.4 Hz, 2H, *α‐H^5^‐Th*), 7.27 (dd, ^3^ *J*_(H,H)_ = 5.1 Hz, ^4^ *J*_(H,H)_ = 1.2 Hz, 1H, *α‐H^5^‐Th*), 7.25–7.21 (m, 4H), 7.06–7.02 (m, 3H), 7.01–6.95 (m, 8H), 6.94–6.87 (m, 6H), 6.83 (dd, ^3^ *J*_(H,H)_ = 3.6 Hz, ^4^ *J*_(H,H)_ = 1.2 Hz, 2H, *β‐H^3^‐Th*), 6.78 (dd, ^3^ *J*_(H,H)_ = 3.7 Hz, ^4^ *J*_(H,H)_ = 1.1 Hz, 1H, *β‐H^3^‐Th*) ppm; HRMS (MALDI‐FTICR) *m*/*z*: calcd. for C_60_H_30_S_10_: 1069.95491; found *m*/*z* = 1069.95351 [M]^+^ (*δm*/*m* = 1.3 ppm), 1321.10808 [M+DCTB]^+^. Isolated derivative B: ^1^H NMR (400 MHz, CD_2_Cl_2_): *δ* 7.57 (dd, ^3^ *J*_(H,H)_ = 5.1 Hz, ^4^ *J*_(H,H)_ = 1.2 Hz, 1H, *α‐H^5^‐Th*), 7.55 (dd, ^3^ *J*_(H,H)_ = 5.1 Hz, ^4^ *J*_(H,H)_ = 1.2 Hz, 1H, *α‐H^5^‐Th*), 7.43 (dd, ^3^ *J*_(H,H)_ = 3.6 Hz, ^4^ *J*_(H,H)_ = 1.2 Hz, 1H, *β‐H^3^‐Th*), 7.38–7.34 (m, 3H), 7.32–7.27 (m, 2H), 7.26–7.17 (m, 6H), 7.05 (dd, ^3^ *J*_(H,H)_ = 3.6 Hz, ^4^ *J*_(H,H)_ = 1.2 Hz, 2H, *β‐H^3^‐Th*), 6.98 (dd, ^3^ *J*_(H,H)_ = 5.1 Hz, ^3^ *J*_(H,H)_ = 3.6 Hz, 2H, *β‐H^4^‐Th*), 6.95–6.94 (m, 2H), 6.93–6.88 (m, 5H), 6.85 (dd, ^3^ *J*_(H,H)_ = 3.6 Hz, ^4^ *J*_(H,H)_ = 1.2 Hz, 1H, *β‐H^3^‐Th*), 6.82 (dd, ^3^ *J*_(H,H)_ = 3.7 Hz, ^4^ *J*_(H,H)_ = 1.2 Hz, 2H, *β‐H^3^‐Th*), 6.73 (dd, ^3^ *J*_(H,H)_ = 5.1, 3.6 Hz, 1H, *β‐H^4^‐Th*), 6.37 (dd, ^3^ *J*_(H,H)_ = 3.6, ^4^ *J*_(H,H)_ = 1.2 Hz, 1H, *β‐H^3^‐Th*) ppm; HRMS (MALDI‐FTICR) *m*/*z*: calcd. for C_60_H_30_S_10_: 1069.95491; found *m*/*z* = 1069.95401 [M]^+^ (*δm*/*m* = 0.8 ppm), 1320.09927 [M+DCTB]^+^.

*1,3,5‐Tris(thien‐2‐yl)‐2,4,6‐tris{[2′‐(thien‐2‐yl)‐3′,4′,5′,6′‐tetraphenyl]benzene}‐benzene* ***9***: Trithiophene **8** (200 mg, 0.31 mmol) and tetracyclone **1** (4.00 g, 10.4 mmol) were stirred at 265 °C in the melt for 24 h. Dendrimer **9** (3%, 16.0 mg, 0.01 mmol) was obtained as colorless solid after purification by column chromatography (PE/DCM 3:1 → 1:1) and purification by HPLC (recycling mode, *n*‐hexane/DCM 4:1). M.p. > 350 °C; ^1^H NMR (500 MHz, TCE‐d_2_): *δ* 7.23 (dd, ^3^ *J*_(H,H)_ = 5.1 Hz, ^4^ *J*_(H,H)_ = 1.1, 2H, *α*‐*H^5′^‐Th*), 7.21 (dd, *^3^J*_(H,H)_ = 5.1 Hz, ^4^ *J*_(H,H)_ = 1.2, 1H, *α*‐*H^5′^‐Th*), 6.89 (dd, ^3^ *J*_(H,H)_ = 5.0 Hz, ^4^ *J*_(H,H)_ = 1.2 Hz, 1H, *α*‐*H^5^‐Th*), 6.88–6.77 (m, 6H, *α*‐*H^5^‐Th, H^2^‐Ph*), 6.76 (dd, ^3^ *J*_(H,H)_ = 5.1, 3.8 Hz, 3H, *β*‐*H^4′^‐Th*), 6.74–6.61 (m, 29H, *H^2^‐Ph, H^3^‐Ph*), 6.59–6.55 (m, 16H, *H^3^‐Ph*), 6.48–6.43 (m, 8H, *β*‐*H^4^‐Th, H^4^‐Ph, H^3^‐Ph*), 6.42 (dd, ^3^ *J*_(H,H)_ = 5.1, 3.8 Hz, 2H, *β*‐*H^4^‐Th*), 6.37–6.35 (m, 7H, *β*‐*H^4^‐Th, H^4^‐Ph, H^3^‐Ph*), 5.38–5.29 (m, 3H, *β*‐*H^4^‐Th*) ppm; ^13^C NMR (126 MHz, TCE‐d_2_): *δ* 142.9, 142.7, 142.3, 142.2, 142.1, 141.5, 141.4, 141.2, 141.2, 140.4, 139.5, 138.5, 138.2, 136.7, 136.7, 132.8, 131.4, 131.2, 130.7, 130.6, 126.8, 126.7, 126.7, 126.6, 126.2, 126.0, 126.0, 125.8, 125.5, 125.2, 125.0, 124.9, 124.9, 124.7, 124.6, 124.2, 123.3 ppm; HRMS (MALDI‐FTICR) *m*/*z*: calcd. for C_120_H_78_S_6_: 1710.44278; found 1710.43980 [M]^+^ (*δm*/*m* = 1.7 ppm), 1734.43117 [M+Na]^+^, 1960.59622 [M+DCTB]^+^; CCDC 1884754.

*2,2′,2″,2‴,2″″‐(3‴,4′,4‴,5′,5‴,6′‐hexaphenyl‐5″‐(thien‐2‐ylethynyl)‐[1,1′:2′,1″:3″,1‴:2‴, 1″″‐quinquephenyl]‐2″,3′,4″,6″,6‴‐pentayl)pentathiophene* ***10*** (54%, 225 mg, 0.17 mmol) was obtained as main product after purification by column chromatography (PE/DCM 3:1 → 1:1). M.p. > 300 °C; ^1^H NMR (400 MHz, CD_3_CN): *δ* 7.44 (dd, ^3^ *J*_(H,H)_ = 5.1, 3.0 Hz, 1H, *β*‐*H^4^‐Th*), 7.38–7.34 (2H, *α*‐*H^5^‐Th*), 7.18 (dd, ^3^ *J*_(H,H)_ = 5.1 Hz, ^4^ *J*_(H,H)_ = 1.1 Hz, 1H, *α*‐*H^5^‐Th*), 6.98–6.91 (m, 3H), 6.90–6.55 (m, 42H), 6.55–6.49 (m, 3H), 6.43 (dd, ^3^ *J*_(H,H)_ = 5.1, 3.6 Hz, 1H, *β*‐*H^4^‐Th*), 6.36 (dd, ^3^ *J*_(H,H)_ = 5.1, 3.6 Hz, 1H, *β*‐*H^4^‐Th*), 5.62–5.58 (m, 2H), 5.54 (dd, ^3^ *J*_(H,H)_ = 3.6, ^4^ *J*_(H,H)_ = 1.2 Hz, 1H, *β*‐*H^3^‐Th*), 5.41 (dd, ^3^ *J*_(H,H)_ = 3.6, ^4^ *J*_(H,H)_ = 1.2 Hz, 1H); ^13^C NMR (500 MHz, CD_2_Cl_2_): *δ* 143.0, 142.9, 142.7, 142.7, 142.7, 142.6, 142.5, 141.7, 141.5, 141.4, 141.2, 140.7, 139.9, 139.7, 139.1, 139.1, 138.2, 138.0, 137.6, 136.0, 132.7, 132.1, 131.9, 131.7, 131.5, 131.3, 131.1, 130.6, 127.5, 126.9, 126.9, 126.8, 126.8, 126.7, 126.6, 126.4, 126.1, 126.1, 125.7, 125.6, 125.6, 124.6, 124.5, 124.5, 124.0, 95.1 ppm (overlapping of signals occurs). HRMS (MALDI‐FTICR) *m*/*z*: calcd. for C_92_H_58_S_6_: 1354.28573; found 1354.28541 [M]^+^ (*δm*/*m* = 0.24 ppm).

*4‐(Thien‐2‐yl)‐6‐(thien‐2‐ylethinyl)‐5‐[2,4,6‐tri(thien‐2‐yl)‐3,5‐bis(thien‐2‐ylethinyl)phenyl]benzo[b]thiophene* **14**: 1,4‐Di(thien‐2‐yl)buta‐1,3‐diyne **7** (200 mg, 0.93 mmol) and [RhCl (PPh_3_)_3_] (86.3 mg, 0.09 mmol) in 20 mL abs. *o*‐xylene were stirred at 130 °C for 3 days. The reaction was stopped by addition of water. The separated organic phase was extracted with DCM and washed with water and dried with sodium sulfate. After removal of the solvent, column chromatography (flash silica gel, PE/DCM 3:2) and HPLC (recycling mode, *n*‐hexane/DCM 3:2) delivered benzo[*b*]thiophene **12** as beige‐colored solid (6.00 mg, 0.007 mmol, 2%). M.p. > 400 °C; ^1^H NMR (400 MHz, CD_2_Cl_2_): *δ* 7.87 (d, ^5^ *J*_(H,H)_ = 0.9 Hz, 1H, *H^5^‐Ph*), 7.58 (dd, ^3^ *J*_(H,H)_ = 5.1 Hz, ^4^ *J*_(H,H)_ = 1.2 Hz, 1H, *α‐H^5^‐Th*), 7.47 (d, ^3^ *J*_(H,H)_ = 5.6 Hz, 1H, *β‐H^4″″^‐Th*) 7.45 (dd, ^3^ *J*_(H,H)_ = 3.6 Hz, *^4^J*_(H,H)_ = 1.2 Hz, 1H, *β‐H^3^‐Th*), 7.40 (dd, ^3^ *J*_(H,H)_ = 4.9 Hz, ^4^ *J*_(H,H)_ = 1.0 Hz, 1H, *α‐H^5^‐Th*), 7.39 (dd, ^3^ *J*_(H,H)_ = 5.3 Hz, ^5^ *J*_(H,H)_ = 0.7 Hz, 1H, *α‐H^5″″^‐Th*), 7.31 (dd, ^3^ *J*_(H,H)_ = 5.1 Hz, ^4^ *J*_(H,H)_ = 1.2 Hz, 1H, *α‐H^5‴^‐Th*), 7.24 (dd, ^3^ *J*_(H,H)_ = 5.1, 3.5 Hz, 1H, *β‐H^4^‐Th*), 7.22 (dd, ^3^ *J*_(H,H)_ = 5.1 Hz, ^4^ *J* = 1.2 Hz, 2H, *α‐H^5′^‐Th*), 7.19 (dd, ^3^ *J*_(H,H)_ = 5.1 Hz, ^4^ *J* = 1.2 Hz, 2H, *α‐H^5″^‐Th*), 7.13 (dd, ^3^ *J*_(H,H)_ = 3.6 Hz, ^4^ *J*_(H,H)_ = 1.2 Hz, 1H, *β‐H^3^‐Th*), 7.06 (dd, ^3^ *J*_(H,H)_ = 5.1, 3.6 Hz, 1H, *β‐H^4^‐Th*), 7.00 (dd, ^3^ *J*_(H,H)_ = 5.2, 3.6 Hz, 1H, *β‐H^4‴^‐Th*), 6.94 (dd, ^3^ *J*_(H,H)_ = 3.6 Hz, ^4^ *J*_(H,H)_ = 1.2 Hz, 2H, *β‐H^3′^‐Th*), 6.88 (dd, ^3^ *J*_(H,H)_ = 5.1, 3.7 Hz, 2H, *β‐H^4′^‐Th*), 6.87 (dd, ^3^ *J*_(H,H)_ = 3.6 Hz, ^4^ *J*_(H,H)_ = 1.2 Hz, 1H, *β‐H^3‴^‐Th*), 6.84 (dd, ^3^ *J*_(H,H)_ = 5.1, 3.6 Hz, 2H, *β‐H^4″^‐Th*), 6.80 (dd, ^3^ *J*_(H,H)_ = 3.7 Hz, ^4^ *J*_(H,H)_ = 1.2 Hz, 2H, *β‐H^3″^‐Th*) ppm; ^13^C NMR (101 MHz, CD_2_Cl_2_): *δ* 141.4, 140.2, 140.0, 139.3, 139.2, 138.9, 138.4, 136.8, 132.6, 132.2, 130.7, 130.3, 130.0, 128.6, 128.6, 128.5, 127.9, 127.6, 127.2, 127.1, 126.9, 126.9, 126.8, 126.2, 126.1, 125.3, 124.2, 123.6, 123.3, 122.4, 94.3, 92.4, 92.2, 88.3 ppm; HRMS (MALDI‐FTICR): *m*/*z* berechnet für C_48_H_34_S_8_: 855.96382; gefunden: 855.96114 [M]^+^ (*δm*/*m* = 3.1 ppm), 1106.10659 [M+DCTB]^+^; CCDC 1884744.

### Computational Methods

*ACID*: The anisotropy of the current density was calculated using ACID^[^ ^17^ ^]^ method implemented in the Gaussian09 D.01^[18]^ program using NMR calculations with continuous set of gauge transformations (CSGT)^[^ ^20^ ^]^ and the IOp(10/93) option. For an adequate description of the expected through‐space conjugation, the Pople basis 6–311+G** with diffuse functions and the standard B3LYP^[^ ^21^ ^]^ density functional was employed. The orientation of the magnetic field vector was orthogonal to the peripheral ring current and pointing toward the viewer.

## Conflict of Interest

The authors declare no conflict of interest.

## Supporting information

Supporting Information

## Data Availability

The data that support the findings of this study are available from the corresponding author upon reasonable request.
