# Supplementary material for: Highly Crowded Twisted Thienylene‐Phenylene Structures: Evidence for Through‐Space Orbital Coupling in a [4]Catenated Topology
Source: Adv Sci (Weinh). 2022 Feb 8;9(19):2105785. doi: 10.1002/advs.202105785 (PMC9259713; doi:10.1002/advs.202105785)
Supplement: Supplementary file 1 — Supporting Information [file ADVS-9-2105785-s001.pdf]

ADVANCED SCIENCE
Open Access

Supporting Information

for *Adv. Sci.*, DOI 10.1002/advs.202105785

Highly Crowded Twisted Thienylene-Phenylene Structures: Evidence for Through-Space Orbital Coupling in a [4]Catenated Topology

*Tanja Desirée Leitner, Jan Simon von Glasenapp, Rainer Herges, Elena Mena-Osteritz and Peter Bäuerle**

ADVANCED SCIENCE
Open Access

Supporting Information

for *Adv. Sci.*, DOI: 10.1002/advs.202105785

Highly crowded twisted thienylene-phenylene structures: Evidence for through-space orbital coupling in a [4]catenated topology

*Tanja Desirée Leitner, Jan Simon von Glasenapp, Rainer Herges, Elena Mena-Osteritz, Peter Bäuerle**

Supporting Information

^{1}H-NMR spectra and high resolution mass spectra (HRMS)

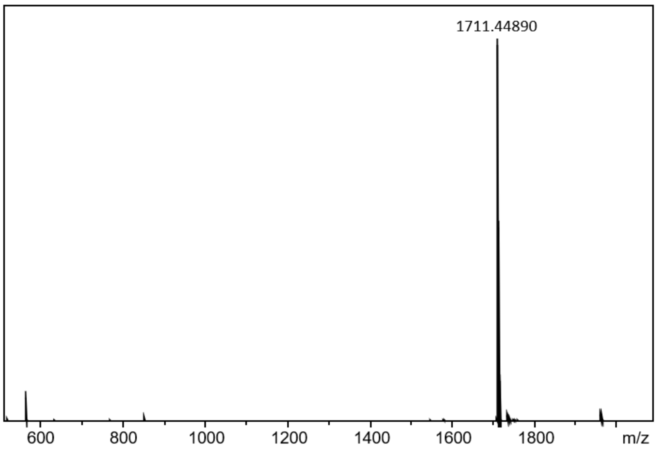

Figure S1 High resolution MALDI FTICR mass spectrum of **9**.

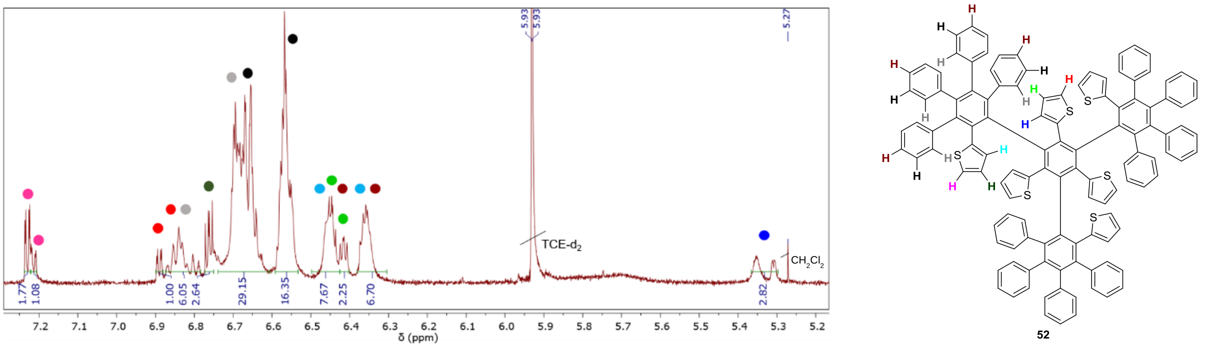

Figure S2 ^{1}H-NMR spectrum of **9** measured in tetrachloroethane-d_2. The colour code is given at the formula (right).

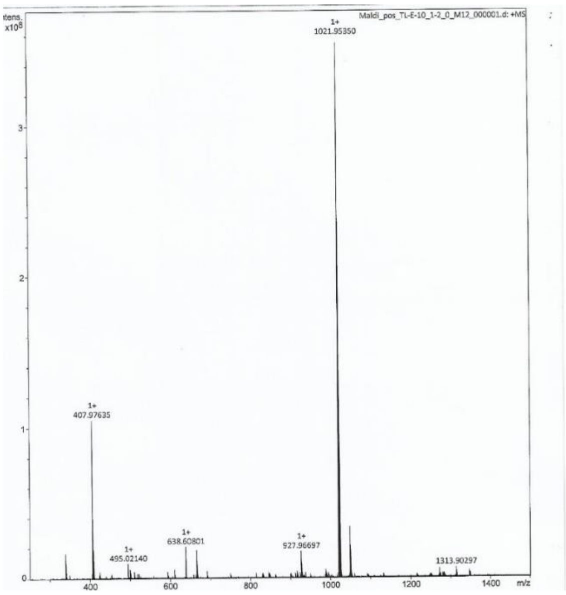

Figure S3 HRMS (MALDI-FTICR) of mono-reacted Diels-Alder adduct **12**; m/z calculated $C_{56}H_{30}S_{10}$: m/z = 1021.95350 [M+] δm/m = 1.4 ppm; m/z = 407.97635 [M+] corresponds to **11**.

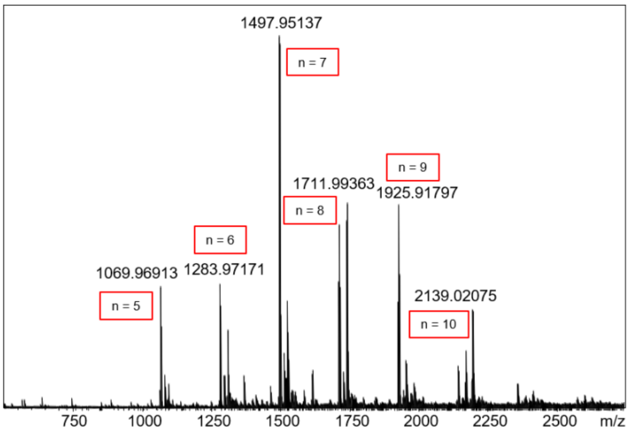

Figure S4 High resolution MALDI FTICR mass spectrum of the raw product of cyclooligomerization of diyne **7**.

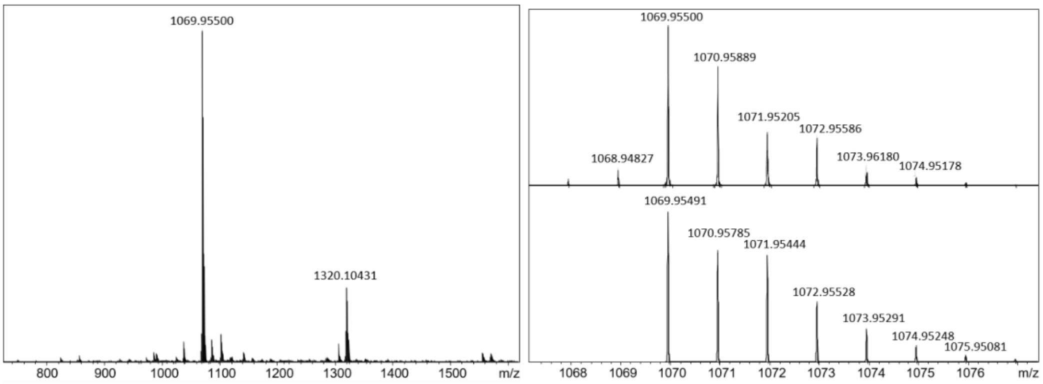

Figure S5 High resolution MALDI FTICR mass spectrum of cyclopentamers **13** (left) and isotopic pattern of the molecular peak (top: experiment, bottom: calculated). The peak at m/z = 1320.1043 corresponds to the mole-cule plus matrix DCTB.

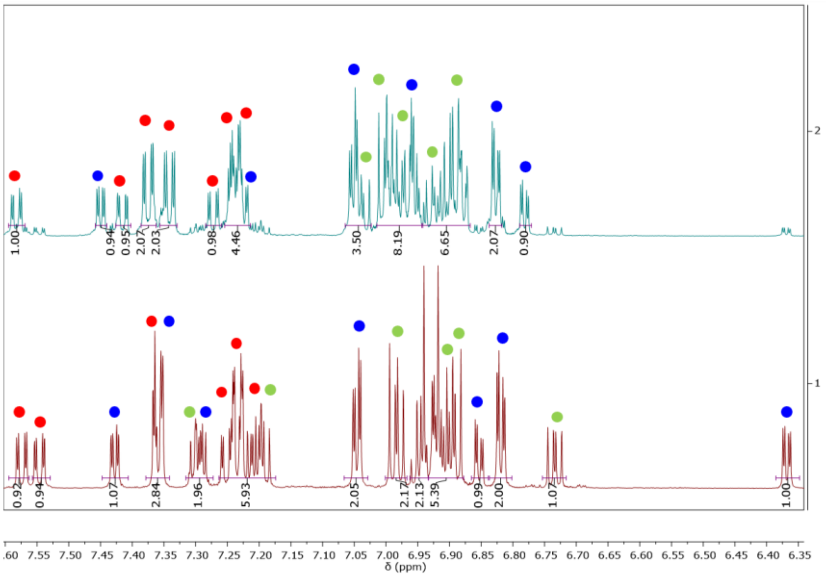

Figure S6 ^{1}H-NMR spectra of HPLC separated fractions of cyclopentamers **13** measured in CD_2Cl_2. Blue circles denote protons in 3-position of thiophenes, green circles in 4-position, and red circles in 5-position.

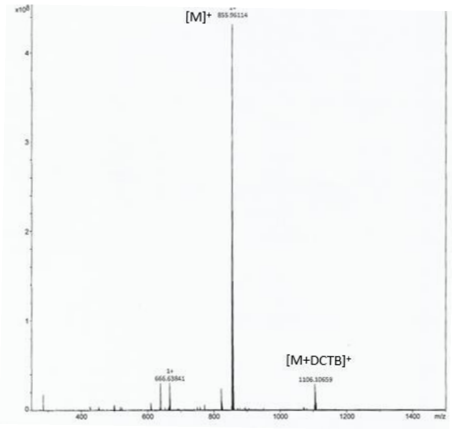

Figure S7 HRMS (MALDI-FTICR) of thienylene-phenylene **14**; m/z calculated $C_{48}H_{34}S_8$: 855.96382; found: 855.96114 $[M]^+$ ($\delta m/m$ = 3.1 ppm), 1106.10659 $[M+DCTB]^+$.

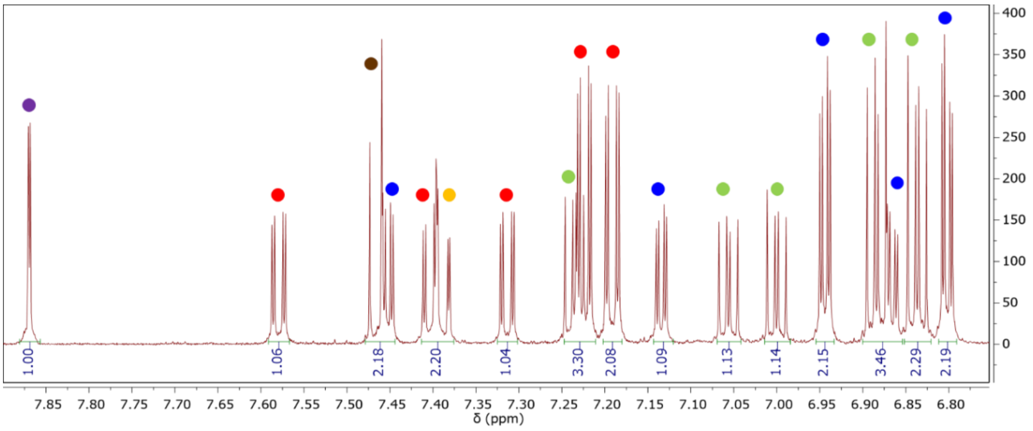

Figure S8 ^{1}H-NMR spectrum of thienylene-phenylene **14** measured in CD_2Cl_2. Blue circles denote protons in 3-position of thiophenes, green circles in 4-position, and red circles in 5-position. The black circles correspond to the signals of the benzo[b]thiophene unit.

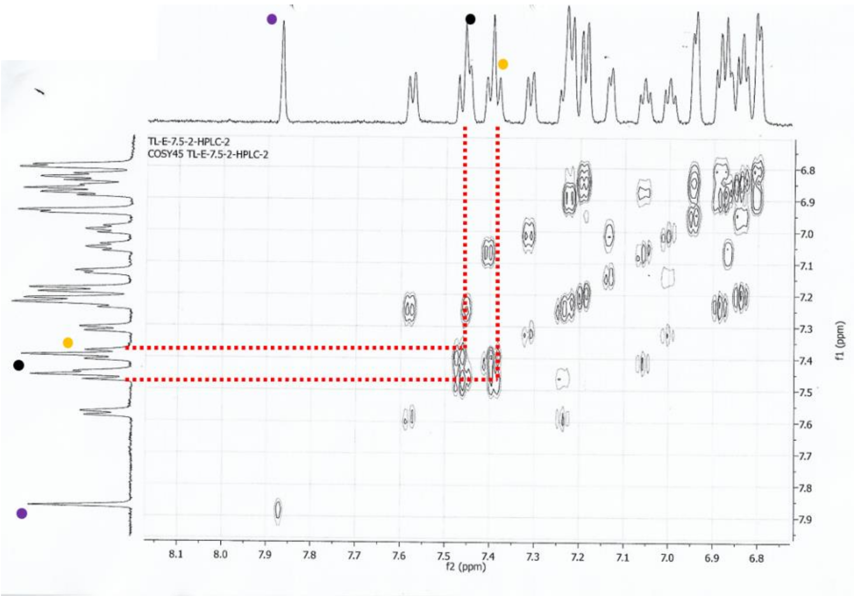

Figure S9 H,H-COSY spectrum of thienylene-phenylene **14** measured in CD_2Cl_2. The black, purple, and yellow circles correspond to the signals of the benzo[b]thiophene unit. Crucial interaction of thiophene protons of benzo[b]thiophene unit is highlighted in red.

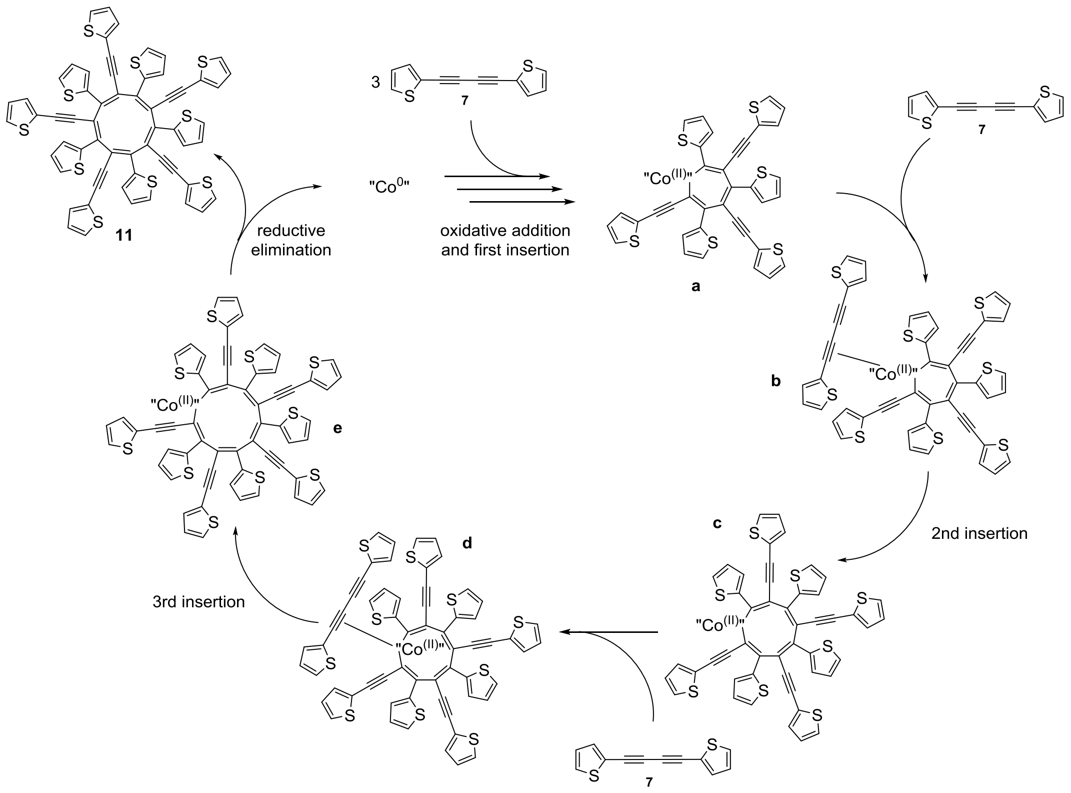

SCHEME S1 Proposed mechanism for the cobalt-mediated formation of cyclopentamers **11** from butadiyne **7**.

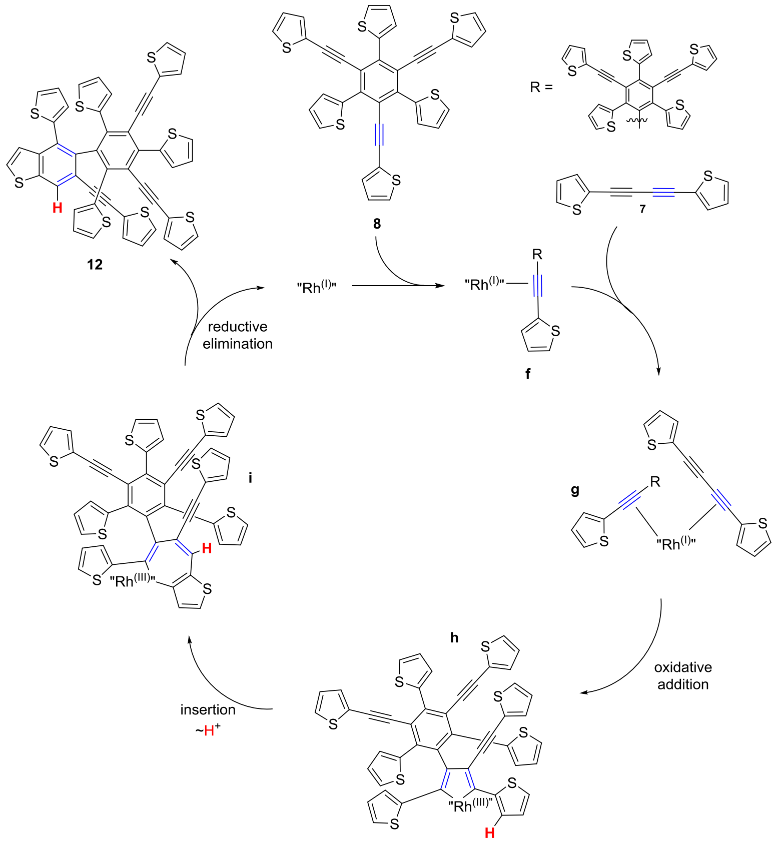

SCHEME S2 Proposed mechanism for the rhodium-mediated formation of benzo[b]thiophene derivative **12** by from thienylene-phenylene **8**.

Single crystal X-ray structure analysis of thienylene-phenylenes 8, 9, and 14

Table S1a Crystallographic data of precursor **8** (CCDC number: 1884748)

Bond precision:	C-C = 0.0030 A	Wavelength= 0.71073	
Cell:	a=50.14(2)	b=7.352(3)	c=16.854(8)
	alpha=90	beta=100.86(2)	gamma=90
Temperature:	293 K		

	Calculated	Reported
Volume	6102(5)	6102(5)
Space group	C 2/c	C 2/c
Hall group	-C 2yc	-C 2yc
Moiety formula	C36 H18 S6	C36 H18 S6
Sum formula	C36 H18 S6	C36 H18 S6
Mr	642.86	642.86
Dx,g cm^{-3}	1.400	1.400
Z	8	8
Mu (mm-1)	0.474	0.474
F000	2640.0	2640.0
F000’	2646.69	
h,k,lmax	58,8,19	58,8,19
Nref	5204	5204
Tmin,Tmax	0.930,0.986	0.700,0.745
Tmin’	0.917	

Correction method= # Reported T Limits: Tmin=0.700 Tmax=0.745
AbsCorr = NONE

Data completeness= 0.999	Theta(max)= 24.687
R(reflections)= 0.0345(4640)	wR2(reflections)= 0.0886(5200)
S = 1.079	Npar= 501

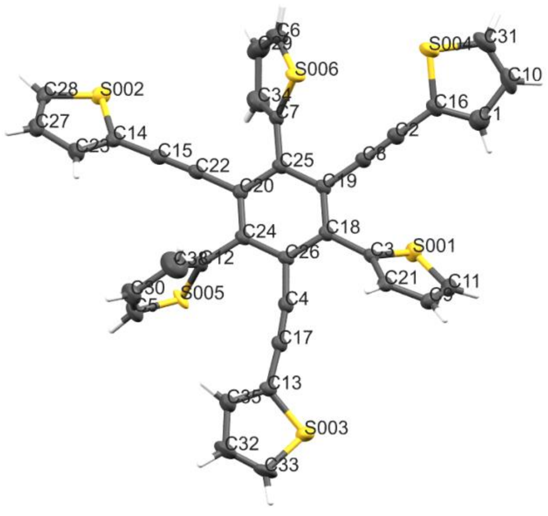

Figure S10a Labelled molecular structure from crystal structure analysis of precursor **8**. For clarity, only one of the two disordered molecules (cis-trans isomerization at certain thiophene rings) is illustrated. Ellipsoids for non-hydrogen atoms are shown at 50% probability.

Table S1b Selected bond distances of precursor **8**.

Atom 1	Atom 2	Length (Å)	Atom 1	Atom 2	Length (Å)
C20	C25	1.411(3)	C20	C22	1.433(3)
C25	C19	1.414(3)	C22	C15	1.201(3)
C19	C18	1.418(2)	C15	C14	1.420(3)
C18	C26	1.417(3)	C14	C23	1.35(1)
C26	C24	1.410(3)	C23	C27	1.41(1)
C24	C20	1.410(3)	C27	C28	1.362(5)
C25	C7	1.483(3)	C28	S002	1.727(4)
C7	C34	1.36(1)	S002	C14	1.722(2)
C34	C29	1.52(1)	C19	C8	1.434(3)
C29	C6	1.335(3)	C8	C2	1.201(3)
C6	S006	1.615(4)	C2	C16	1.423(3)
S006	C7	1.693(4)	C16	C1	1.38(1)
C18	C3	1.479(3)	C1	C10	1.44(1)
C3	C21	1.392(3)	C10	C31	1.331(4)
C21	C9	1.419(3)	C31	S004	1.668(3)
C9	C11	1.354(3)	S004	C16	1.707(3)
C11	S001	1.714(2)	C26	C4	1.437(3)
S001	C3	1.736(2)	C4	C17	1.204(3)
C24	C12	1.486(3)	C17	C13	1.421(3)
C12	C38	1.25(1)	C13	C35	1.36(1)
C38	C30	1.53(1)	C35	C32	1.42(1)
C30	C5	1.333(4)	C32	C33	1.36(1)
C5	S005	1.667(3)	C33	S003	1.724(5)
S005	C12	1.715(3)	S003	C13	1.714(3)

Table S1c Selected angles of precursor **8**.

Atom 1	Atom 2	Atom 3	Angle (deg.)
C24	C26	C4	116.5(2)
C26	C4	C17	170.9(2)
C4	C17	C13	175.5(2)
C17	C13	C35	126.0(6)
C24	C20	C22	118.9(2)
C20	C22	C15	177.2(2)
C22	C15	C14	178.4(2)
C15	C14	C23	127.3(5)
C18	C19	C8	120.7(2)
C19	C8	C2	177.9(2)
C8	C2	C16	179.5(2)
C2	C16	C1	129.5(5)

Table S1d Selected torsion angles of precursor **8**.

Atom 1	Atom 2	Atom 3	Atom 4	Torsion (deg.)
C19	C18	C3	S001	-48.0(2)
C19	C25	C7	S006	-63.0(3)
C26	C24	C12	S005	-81.3(2)
C25	C19	C16	S004	18.4(2)
C25	C20	C14	S002	27.1(2)
C18	C26	C13	S003	-18.9(2)

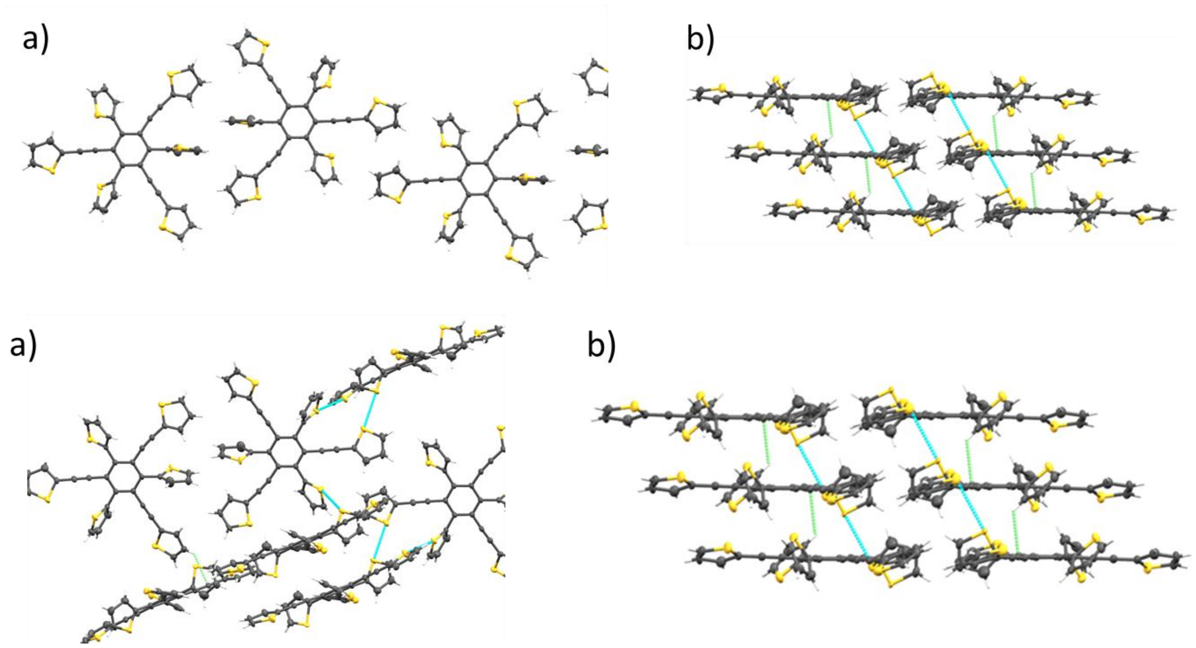

Figure S10b Packing mode of **8** perpendicular (a) and parallel (b) to the [-5 2 7] plane. Intermolecular interactions have been analysed (see also table S1e): S-S and C-H atomic contacts at distances below van der Waals radii are labelled with cyan and green dotted lines, respectively. Distances between the planes in b) are 4.045 Å.

Table S1e Intermolecular short contacts, below van der Waals radii, of **8**.

Atom Mol. 1	Atom Mol. 2	Length (Å)	Mol. 1	Mol. 2
S003	S005	3.543	x,y,z	x,-1+y,z
H21	C22	2.807	x,y,z	x,-1+y,z
S004	S006	3.288	x,y,z	1-x,y,1/2-z
S006	S001	3.554	x,y,z	x,1-y,-1/2+z
H27	C17	2.830	x,y,z	x,2-y,-1/2+z

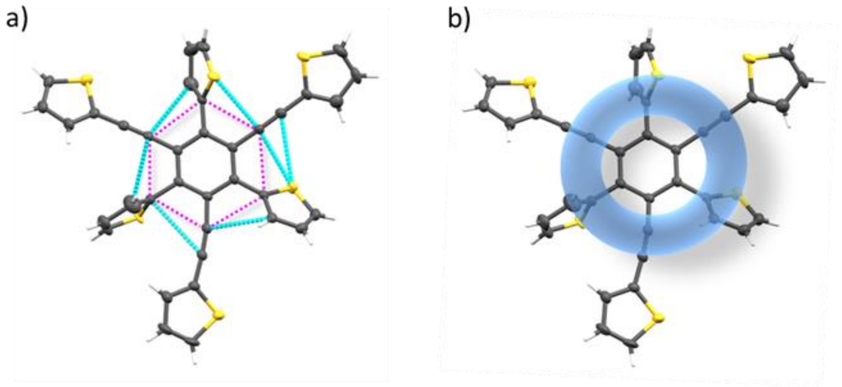

Figure S10c Intramolecular short contacts in hexasubstituted benzene derivative **8** (see also table S1f): Short atomic (C-C and C-S) contacts below van der Waals radii are labelled in cyan. The interactions between ipso-C atoms are highlighted with magenta lines (a). The toroidal short-contact topology is sketched in (b).

Table S1f Intramolecular short contacts, below van der Waals radii, of **8**.

Atom 1	Atom 2	Length (Å)
C8	C3	2.923
C3	C4	2.933
C4	C12	2.814
C12	C22	2.827
C22	C7	2.871
C7	C8	2.864
S001	C2	3.455
S001	C8	3.111
S006	C8	3.234
C4	C21	3.034
C12	C17	3.382
C22	C34	3.196
C22	C38	3.338

Table S2a Crystallographic data of of **9** (CCDC number: 1884754)

Bond precision:	C-C = 0.0042 Å	Wavelength=1.54184	
Cell:	a=13.0074(6)	b=18.8041(7)	c=20.8851(9)
	alpha=104.484(4)	beta=97.403(4)	gamma=92.008(4)
Temperature:	150 K		

	Calculated	Reported
Volume	4892.7(4)	4892.7(4)
Space group	P -1	P -1
Hall group	-P 1	-P 1
Moiety formula	C119.97 H77.97 S6 [+solvent]	C119.97 H77.97 S5.99
Sum formula	C119.97 H77.97 S6 [+solvent]	C119.97 H77.97 S5.99
Mr	1711.55	1711.47
Dx,g (cm^{-3})	1.162	1.162
Z	2	2
Mu (mm^{-1})	1.660	1.660
F000	1787.3	1787.0
F000’	1795.49	
h,k,lmax	16,23,26	16,23,26
Nref	19951	19155
Tmin,Tmax	0.773,0.847	0.621,1.000
Tmin’	0.694	

Correction method= # Reported	T Limits: Tmin=0.621 Tmax=1.000
AbsCorr = MULTI-SCAN	
Data completeness= 0.960	Theta(max)= 74.256
R(reflections)= 0.0808(13890)	wR2(reflections)= 0.2644(19155)
S = 1.106	Npar= 1960

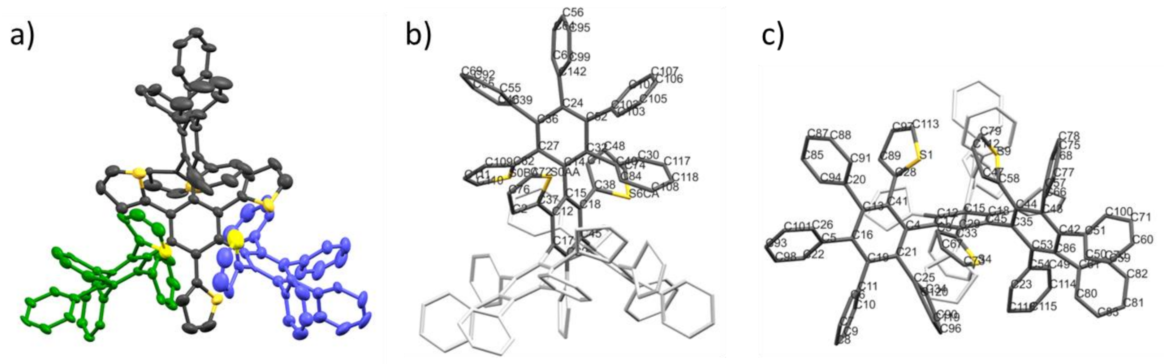

Figure S11 Molecular structure of dendrimer **9** with ellipsoids at 50% probability for heavy atoms (a): each penta(hetero)arylphenyl unit has been distinctly coloured. Labelled molecular structure of **9** depicted in two images for clarity: central phenyl ring with two 2-thienyl groups and one penta(hetero)arylphenyl unit (b) and central phenyl ring with the remaining two penta(hetero)arylphenyl units and 2-thienyl group (c). For clarity also, only one of the two disordered molecules (cis-trans isomerization at certain thiophene rings) is illustrated and hydrogen atoms and ellipsoids have been avoided.

Table S2b Selected bond distances of **9**.

Atom 1	Atom 2	Length (Å)	Atom 1	Atom 2	Length (Å)
C12	C37	1.486(4)	C24	C36	1.47(2)
C29	C33	1.490(4)	C52	C24	1.35(2)
C18	C38	1.507(5)	C32	C52	1.50(2)
C17	C4	1.506(5)	C14	C32	1.34(2)
C45	C35	1.515(4)	C17	C12	1.415(4)
C15	C14	1.501(4)	C29	C17	1.417(4)
C53	C35	1.47(2)	C45	C29	1.420(5)
C86	C53	1.33(2)	C18	C45	1.410(4)
C42	C86	1.45(2)	C15	C18	1.412(4)
C43	C42	1.31(2)	C12	C15	1.416(5)
C44	C43	1.51(1)	C33	C3	1.32(1)
C35	C44	1.35(1)	C3	C67	1.54(2)
C21	C4	1.394(4)	C67	C73	1.34(3)
C19	C21	1.47(2)	C73	S4	1.50(2)
C16	C19	1.35(3)	S4	C33	1.742(5)
C13	C16	1.47(3)	C38	C1	1.31(2)
C41	C13	1.32(4)	C1	C48	1.48(3)
C4	C41	1.47(2)	C48	C74	1.34(3)
C27	C14	1.47(2)	C74	S6CA	1.72(2)
C36	C27	1.31(2)	S6CA	C38	1.788(5)

Table S2c Selected torsion angles of **9**.

Atom 1	Atom 2	Atom 3	Atom 4	Torsion (deg.)
C15	C12	C37	S0AA	-40.5(4)
C45	C29	C33	S4	-45.0(4)
C45	C18	C38	S6CA	-43.1(4)
C29	C17	C4	C21	-71.9(4)
C4	C41	C28	S1	-80(2)
C41	C13	C20	C91	-70(2)
C13	C16	C5	C26	-57(3)
C16	C19	C11	C6	-59(2)
C19	C21	C25	C120	-66.5(8)
C29	C45	C35	C44	-66.5(8)
C35	C44	C58	S9	-63(2)
C44	C43	C57	C68	-68(1)
C43	C42	C51	C100	-63(2)
C42	C86	C61	C70	-65(2)
C86	C53	C54	C49	-62(3)
C12	C15	C14	C27	-76.7(9)
C14	C27	C62	S0BA	-81(1)
C27	C36	C39	C46	-51(3)
C36	C24	C142	C63	-66(2)
C24	C52	C102	C104	-67(2)
C52	C32	C40	C30	-65(3)

Table S2d Intermolecular short contacts, below van der Waals radii, of **9**.

Atom Mol. 1	Atom Mol. 2	Length	Mol. 1	Mol. 2
C97	H96	2.736	x,y,z	-1+x,y,z
H118	C83	2.776	x,y,z	-1+x,y,z
H98	C75	2.887	x,y,z	x,-1+y,z
C22	H95	2.796	x,y,z	-x,-y,1-z
H30	H117	2.387	x,y,z	-x,1-y,1-z
H68	H107	2.309	x,y,z	-x,1-y,1-z
C117	H117	2.880	x,y,z	-x,1-y,1-z
H117	H117	2.261	x,y,z	-x,1-y,1-z
H8	C93	2.854	x,y,z	1-x,-y,2-z
C48	C82	3.395	x,y,z	1-x,1-y,1-z
H48	C82	2.653	x,y,z	1-x,1-y,1-z
H63	C82	2.857	x,y,z	1-x,1-y,1-z
H74	H114	2.347	x,y,z	1-x,1-y,1-z

Table S2e Intramolecular short contacts, below van der Waals radii, between *ipso*-C atoms of **9**.

Atom1	Atom2	Length (Å)
C62	C39	2.93(2)
C39	C142	2.74(3)
C142	C102	3.09(3)
C102	C40	2.84(2)
C40	C15	2.95(2)
C15	C62	2.939(6)
C14	C37	2.908(5)
C37	C4	2.900(4)
C4	C33	2.907(4)
C33	C35	2.919(5)
C35	C38	2.942(4)
C38	C14	2.921(4)
C45	C54	3.04(3)
C54	C61	2.87(3)
C61	C51	2.81(1)
C51	C57	3.00(1)
C57	C58	2.81(2)
C58	C45	2.93(2)
C17	C28	3.00(3)
C28	C20	2.72(4)
C20	C5	3.02(3)
C5	C11	2.92(4)
C11	C25	2.839(8)
C17	C25	2.985(4)

Table S2f Intramolecular short contacts, below van der Waals radii, of **9** excluding contacts between *ipso*-C atoms.

Atom 1	Atom 2	Length (Å)	Atom 1	Atom 2	Length (Å)
C1	C14	2.976	C38	C54	3.209
C1	C32	3.258	C39	C63	2.956
C1	C49	3.162	C39	C109	3.216
C2	C4	2.926	C40	C103	3.272
C2	C21	3.391	C46	C62	3.118
C2	C41	3.270	C47	C57	3.020
C3	C4	2.942	C47	C68	3.350
C3	C21	3.158	C49	C61	3.033
C3	C120	3.366	C50	C61	3.298
C5	C6	3.142	C51	C66	3.199
C5	C94	3.175	C51	C70	3.127
C6	C22	3.336	C54	C80	3.191
C10	C25	3.116	C55	C63	3.361
C11	C22	3.032	C55	C142	2.941
C11	C120	3.009	C57	C100	3.205
C12	C28	3.302	C58	C68	3.093
C12	C62	3.306	C73	C120	3.364
C15	C84	3.262	C84	C103	3.318
C17	C34	3.284	C89	C94	3.328
C18	C40	3.257	C99	C102	3.032
C18	C54	3.298	C99	C104	3.231
C20	C26	3.136	C108	C113	3.243
C20	C89	3.054	S0AA	C14	2.962
C21	C33	3.241	S0AA	C27	3.438
C23	C45	3.390	S0AA	C32	3.176
C25	C29	3.333	S0AA	C89	3.241
C25	C33	3.248	S4	C35	2.980
C27	C37	3.383	S4	C44	3.271
C28	C37	3.244	S4	C53	3.329
C28	C91	3.059	S4	C120	3.258
C29	C58	3.277	S9	C45	3.415
C30	C38	3.175	S9	C108	3.351
C30	C102	3.226	S9	C118	3.413
C32	C38	3.276	C15	S0BA	3.420
C33	C44	3.171	C30	S6CA	3.144
C33	C58	3.298	C35	S6CA	2.955
C33	C120	3.275	C44	S6CA	3.298
C37	C41	3.305	C53	S6CA	3.346
C37	C62	3.340	C79	S1	3.282
C37	C89	3.344	C91	S1	3.469
C38	C40	3.182	S1	C112	3.378
C38	C49	3.239	S0BA	C115	3.326
C38	C53	3.291	S0BA	C116	3.235

Table S3a Crystallographic data of **14** (CCDC number: 1884744)

Bond precision:	C-C = 0.0030 A	Wavelength=1.54184	
Cell:	a=9.5703(4)	b=11.4821(4)	c=19.3739(5)
	alpha=102.118(3)	beta=96.605(3)	gamma=98.016(3)
Temperature:	150 K		
	Calculated	Reported	
Volume	2038.14(13)	2038.14(12)	
Space group	P -1	P -1	
Hall group	-P 1	-P 1	
Moiety formula	C48 H24 S8	C48 H24 S8	
Sum formula	C48 H24 S8	C48 H24 S8	
Mr	857.15	857.15	
Dx,g cm^{-3}	1.397	1.397	
Z	2	2	
Mu (mm-1)	4.327	4.327	
F000	880.0	880.0	
F000’	887.04		
h,k,lmax	11,14,24	11,14,23	
Nref	8291	8009	
Tmin,Tmax	0.390,0.510	0.487,0.653	
Tmin’	0.295		

Correction method= # Reported T Limits: Tmin=0.487 Tmax=0.653
AbsCorr = GAUSSIAN

Data completeness= 0.966	Theta(max)= 74.214
R(reflections)= 0.0457(7227)	wR2(reflections)= 0.1303(8009)
S = 1.026	Npar= 727

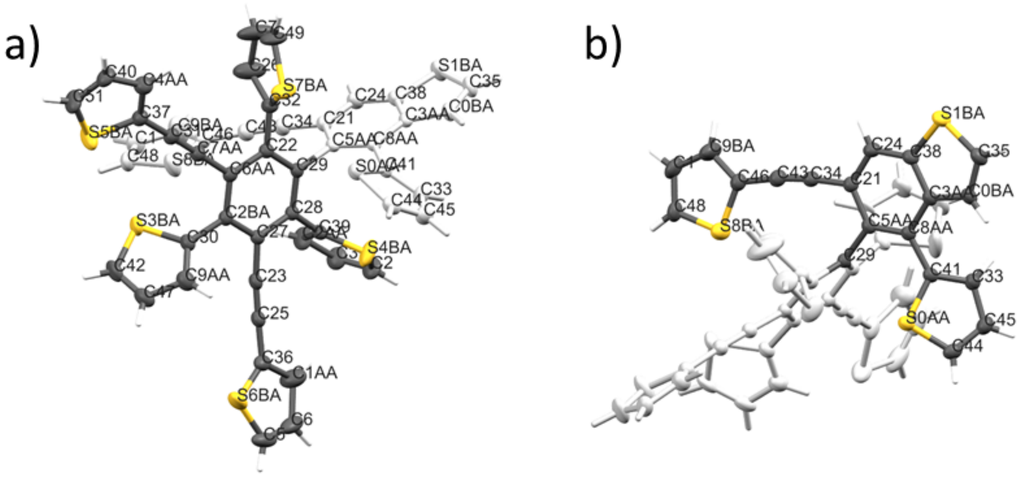

Figure S12a Labelled molecular structure from the crystal structure analysis of compound **14** depicted in two images for clarity: central phenyl ring with three 2-thienyl and two 2-thienylethynyl groups (a) and the 7-benzo-[b]thiophene unit (b). For clarity also, only one of the two disordered molecules (cis-trans isomerization at certain thiophene rings) is illustrated. Ellipsoids for non-hydrogen atoms are shown at 50% probability.

Table S3b Selected bond distances of **14**.

Atom 1	Atom 2	Length (Å)	Atom 1	Atom 2	Length (Å)
C2BA	C27	1.407(3)	C23	C25	1.196(3)
C27	C28	1.414(3)	C25	C36	1.408(3)
C28	C29	1.400(2)	C36	C1AA	1.56(2)
C29	C22	1.401(3)	C1AA	C6	1.47(1)
C22	C6AA	1.414(3)	C6	C5	1.34(1)
C6AA	C2BA	1.412(3)	C5	S6BA	1.702(8)
C2BA	C30	1.480(3)	S6BA	C36	1.626(4)
C30	C9AA	1.345(6)	C29	C5AA	1.494(3)
C9AA	C47	1.41(1)	C5AA	C21	1.424(3)
C47	C42	1.36(1)	C21	C24	1.386(3)
C42	S3BA	1.712(5)	C24	C38	1.388(3)
S3BA	C30	1.736(3)	C38	C3AA	1.412(3)
C28	C39	1.478(3)	C3AA	C8AA	1.426(3)
C39	C2AA	1.47(2)	C8AA	C5AA	1.397(3)
C2AA	C3	1.46(2)	C5AA	C21	1.424(3)
C3	C2	1.335(9)	C3AA	C0BA	1.433(3)
C2	S4BA	1.710(6)	C0BA	C35	1.351(3)
S4BA	C39	1.661(3)	C35	S1BA	1.722(2)
C22	C32	1.483(3)	S1BA	C38	1.730(2)
C32	C26	1.452(8)	C8AA	C41	1.481(3)
C26	C7	1.44(1)	C41	C33	1.365(3)
C7	C49	1.34(2)	C33	C45	1.419(3)
C49	S7BA	1.69(1)	C45	C44	1.350(4)
S7BA	C32	1.670(4)	C44	S0AA	1.715(2)
C6AA	C7AA	1.430(3)	S0AA	C41	1.736(2)
C7AA	C31	1.196(3)	C21	C34	1.429(3)
C31	C37	1.413(3)	C34	C43	1.198(3)
C37	C4AA	1.357(8)	C43	C46	1.417(3)
C4AA	C40	1.41(1)	C46	C9BA	1.38(1)
C40	C51	1.35(1)	C9BA	C1	1.40(3)
C51	S5BA	1.715(9)	C1	C48	1.36(3)
S5BA	C37	1.715(3)	C48	S8BA	1.71(2)
C27	C23	1.429(3)	S8BA	C46	1.683(4)

Table S3c Selected bond angles of **14**.

Atom 1	Atom 2	Atom 3	Angle (deg.)
C2BA	C6AA	C7AA	119.7(2)
C6AA	C7AA	C31	178.6(2)
C7AA	C31	C37	177.0(2)
C28	C27	C23	118.1(2)
C27	C23	C25	173.7(2)
C23	C25	C36	178.0(2)
C5AA	C21	C34	118.1(2)
C21	C34	C43	174.3(2)
C34	C43	C46	173.8(2)

Table S3d Selected torsion angles of **14**.

Atom 1	Atom 2	Atom 3	Atom 4	Torsion (deg.)
C29	C28	C39	S4BA	83.3(3)
C6AA	C2BA	C30	S3BA	55.5(3)
C6AA	C22	C32	S7BA	76.1(3)
C2BA	C6AA	C37	S5BA	-25.4(2)
C2BA	C27	C36	S6BA	-17.1(3)
C22	C29	C5AA	C21	-85.3(2)
C5AA	C8AA	C41	S0AA	-49.3(3)
C5AA	C21	C46	S8BA	7.0(2)

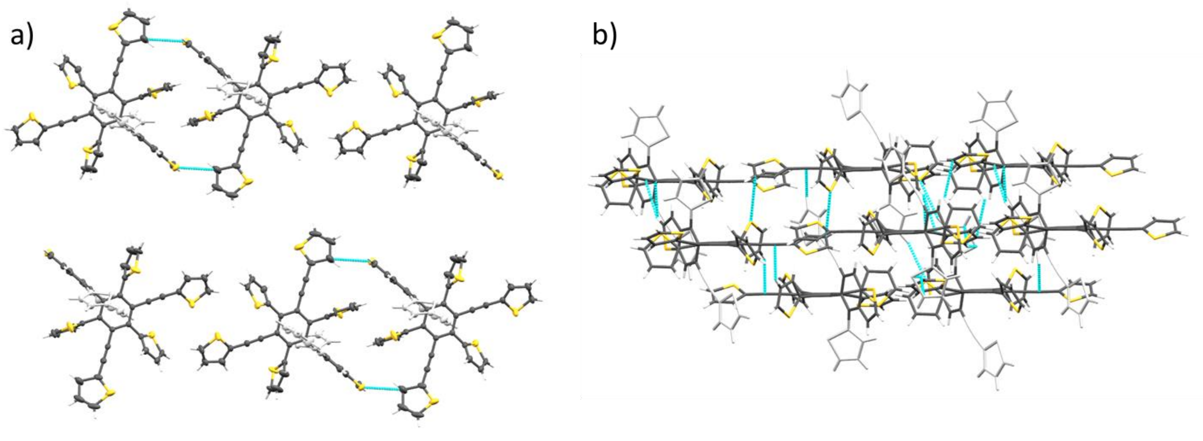

Figure S12b Packing mode of **14**: perpendicular (a) and parallel (b) to the [9 -4 4] plane. Intermolecular contacts observed between atoms at distances below van der Waals radii are labelled by dotted cyan lines (see also table S3e). The out-of-plane pending 7-benzo[b]thiophene units have been foggy up for clarity. Ellipsoids for non-hydrogen atoms are shown at 50% probability in a) and are omitted in b) for clarity.

Table S3e Intermolecular short contacts, below van der Waals radii, of **14**.

Atom 1	Atom 2	Length (Å)	Mol. 1	Mol. 2
H1	C31	2.786	x,y,z	-1+x,y,z
H35	C23	2.753	x,y,z	x,-1+y,z
H35	C25	2.886	x,y,z	x,-1+y,z
H7	C31	2.753	x,y,z	-x,-y,-1-z
S1BA	C1AA	3.491	x,y,z	-x,-y,-z
S1BA	H1AA	2.891	x,y,z	-x,-y,-z
H2AA	C6	2.858	x,y,z	-x,1-y,-z
S7BA	C40	3.457	x,y,z	1-x,-y,-1-z
S7BA	H40	2.962	x,y,z	1-x,-y,-1-z
S4BA	H45	2.984	x,y,z	1-x,-y,-z
S0AA	H5	2.935	x,y,z	1-x,1-y,-z
H5	C44	2.847	x,y,z	1-x,1-y,-z

Table S3f Intramolecular short contacts, below van der Waals radii, of **14**.

Atom 1	Atom 2	Length (Å)
C30	C23	2.894
C23	C39	2.802
C39	C5AA	2.888
C5AA	C32	2.962
C32	C7AA	2.815
C7AA	C30	2.893
S0AA	S7BA	3.402
S0AA	C22	3.409
S0AA	C29	3.215
S3BA	C7AA	3.176
S3BA	S8BA	3.523
C26	C7AA	3.399
C0BA	C33	3.174
C2AA	C23	3.250
C7AA	S7BA	3.419
C9AA	C23	3.065
C22	C34	3.290
C28	C34	3.340
C29	C43	3.368

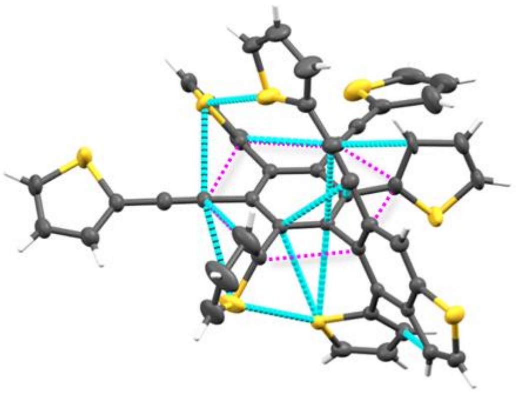

Figure S12c Intramolecular short contacts in **14** (see also table S3f): Short atomic (C-C and C-S) contacts below van der Waals radii are labelled in cyan. The interactions between *ipso*-C atoms are highlighted with magenta lines.

Electrochemistry of thienylene-phenylenes 8 and 9

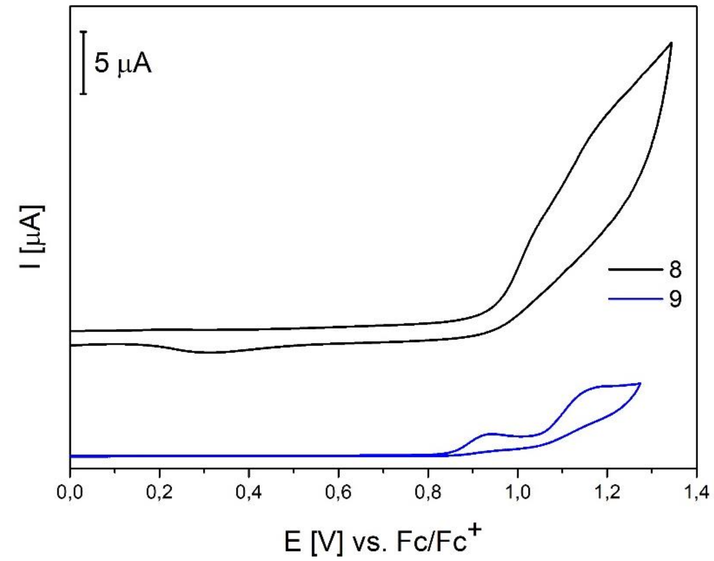

Figure S13 Cyclic voltammograms of precursor **8** (black curve) and dendrimer **9** (blue curve) measured in DCM at r.t., tetrabutylammonium hexafluorophosphate ($TBAPF_6$, 0.1 M), scan speed 100 mV/s, c= $5{\cdot}10^{-4}$ mol/L, potentials vs ferrocene/ferricenium (Fc/Fc^+).
